# Supplementary material for: Active site remodeling in tumor-relevant IDH1 mutants drives distinct kinetic features and potential resistance mechanisms
Source: Nat Commun. 2024 May 6;15:3785. doi: 10.1038/s41467-024-48277-2 (PMC11074275; doi:10.1038/s41467-024-48277-2)
Supplement: Supplementary file 1 — Supplementary Information [file 41467_2024_48277_MOESM1_ESM.pdf]

Active site remodeling in tumor-relevant IDH1 mutants drives distinct kinetic features and potential resistance mechanisms

Matthew Mealka[1], Nicole A. Sierra[1], Diego Avellaneda Matteo[1], Elene Albekioni[1], Rachel Khoury[1], Timothy Mai[1], Brittany M. Conley[1], Nalani J. Coleman[1], Kaitlyn A. Sabo[1], Elizabeth A. Komives[2], Andrey A. Bobkov[3], Andrew L. Cooksy[1], Steve Silletti[2], Jamie M. Schiffer[4], Tom Huxford[1], and Christal D. Sohl[1,*]

Supplementary Information Table of Contents:

Supplementary Figs. 1-19

Supplementary Tables 1-6

Supplementary References

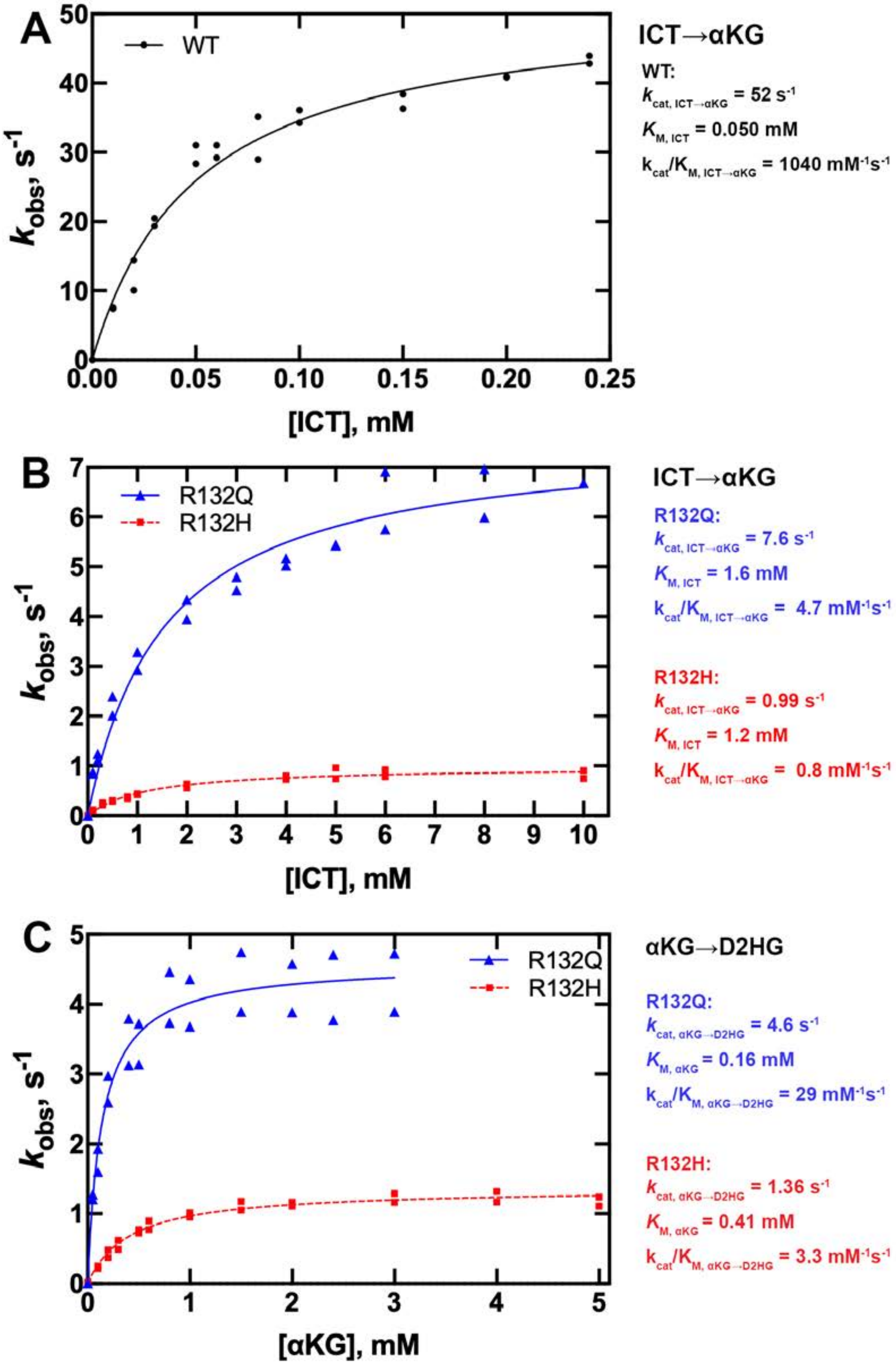

Supplementary Fig. 1. Steady-state kinetics analyses for IDH1 WT, R132Q, and R132H homodimers. Steady-state kinetic parameters of the conventional and neomorphic reactions were measured as a function of varying substrate concentration. Four biological replicates via protein preparations were used to characterize neomorphic reaction kinetic parameters for R132Q, while three biological replicates via protein preparations were used in all other cases. Replicate points are shown. Observed rate constants (k_{obs}) were determined from the linear portion of plots of substrate concentration versus time. A) The conventional reaction catalyzed by IDH1 WT in black circles. The following kinetic parameters were measured for IDH1 WT: $k_{cat,\ ICT\rightarrow\alpha KG}$ = 52 ± 2 s^{-1}; $K_{m,\ ICT}$ = 0.050 ± 0.005 mM; $k_{cat}/K_{m,\ ICT\rightarrow\alpha KG}$ = 1040 ± 110 mM^{-1} s^{-1}. B) The conventional reaction catalyzed by

IDH1 R132Q (blue triangles) and IDH1 R132H (red squares). The following kinetic parameters were measured for IDH1 R132Q: $k_{cat,\ ICT \rightarrow \alpha KG}$ = 7.6 ± 0.3 s^{-1}; $K_{m,\ ICT}$ = 1.6 ± 0.2 mM; $k_{cat}/K_{m,\ ICT \rightarrow \alpha KG}$ = 4.7 ± 0.6 mM^{-1} s^{-1}. The following kinetic parameters were measured for IDH1 R132H: $k_{cat,\ ICT \rightarrow \alpha KG}$ = 0.99 ± 0.04 s^{-1}; $K_{m,\ ICT}$ = 1.2 ± 0.2 mM; $k_{cat}/K_{m,\ ICT \rightarrow \alpha KG}$ = 0.8 ± 0.1 mM^{-1} s^{-1}. C) The neomorphic reaction catalyzed by IDH1 R132Q (blue triangles) and IDH1 R132H (red squares). The following kinetic parameters were measured for IDH1 R132Q: $k_{cat,\ \alpha KG \rightarrow D2HG}$ = 4.6 ± 0.1 s^{-1}; $K_{m,\ \alpha KG}$ = 0.16 ± 0.01 mM; $k_{cat}/K_{m,\ \alpha KG \rightarrow D2HG}$ = 29 ± 2 mM^{-1} s^{-1}. The following kinetic parameters were measured for IDH1 R132H: $k_{cat,\ \alpha KG \rightarrow D2HG}$ = 1.36 ± 0.03 s^{-1}; $K_{m,\ \alpha KG}$ = 0.41 ± 0.04 mM; $k_{cat}/K_{m,\ \alpha KG \rightarrow D2HG}$ = 3.3 ± 0.3 mM^{-1} s^{-1}. Kinetic parameters were calculated and reported as +/- SEM resulting from deviation of the mathematical fit.

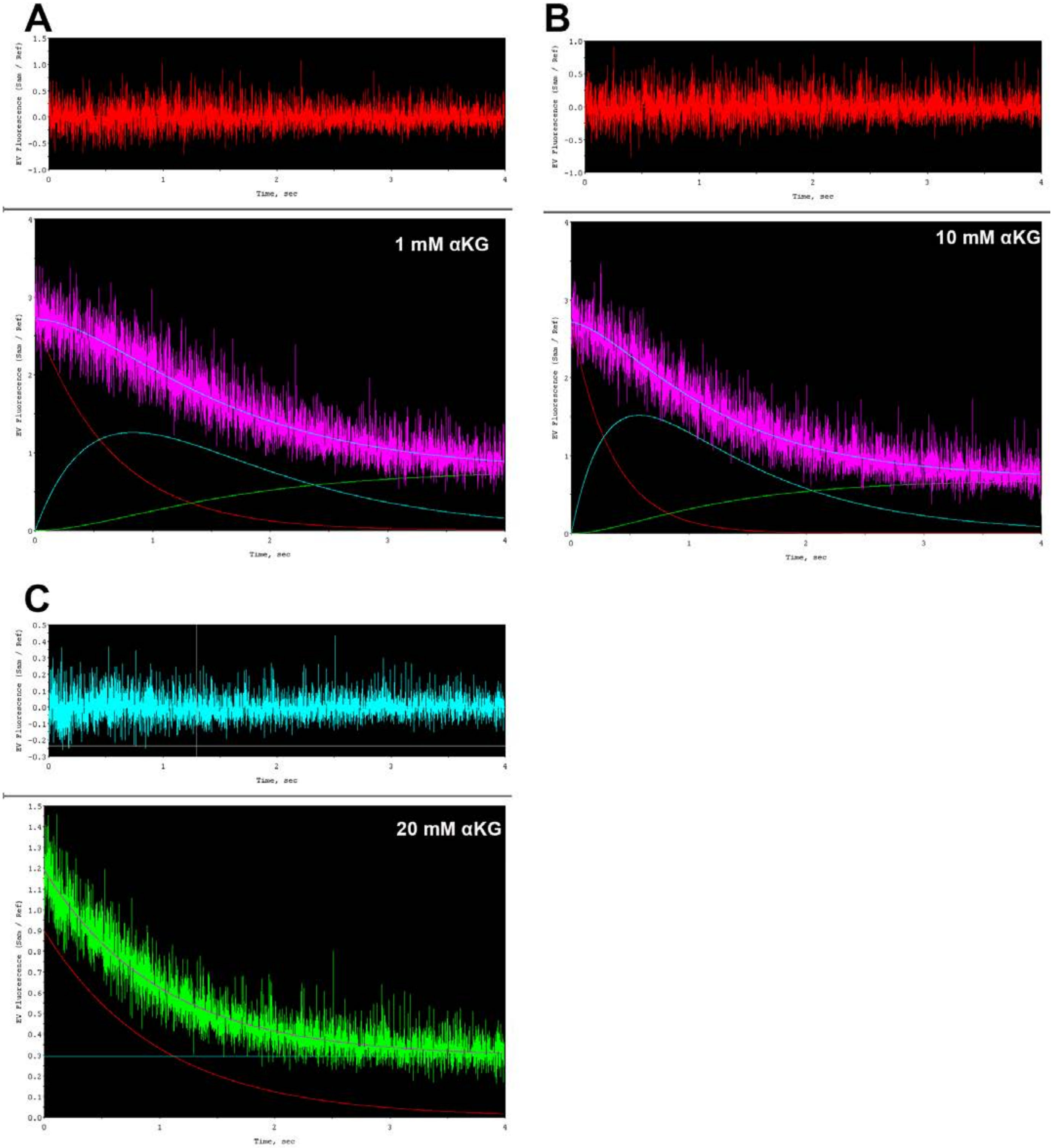

Supplementary Fig. 2. Lag formation when monitoring hydride transfer by IDH1 R132H upon catalyzing the neomorphic reaction. A) A notable lag in NADPH consumption during IDH1 R132H catalysis was seen upon treatment of 1 mM αKG. B) The lag was lessened upon increasing substrate concentration to 10 mM αKG. C) The lag was finally eliminated upon using 20 mM αKG. Data in (A) and (B) were fit to a double exponential equation and data in (C) were fit to a single exponential equation, with the residuals shown (top of each plot) to assess goodness of fit. In all cases, a single protein preparation (biological replicate) was used, with each trace representing an average of 4 technical replicates.

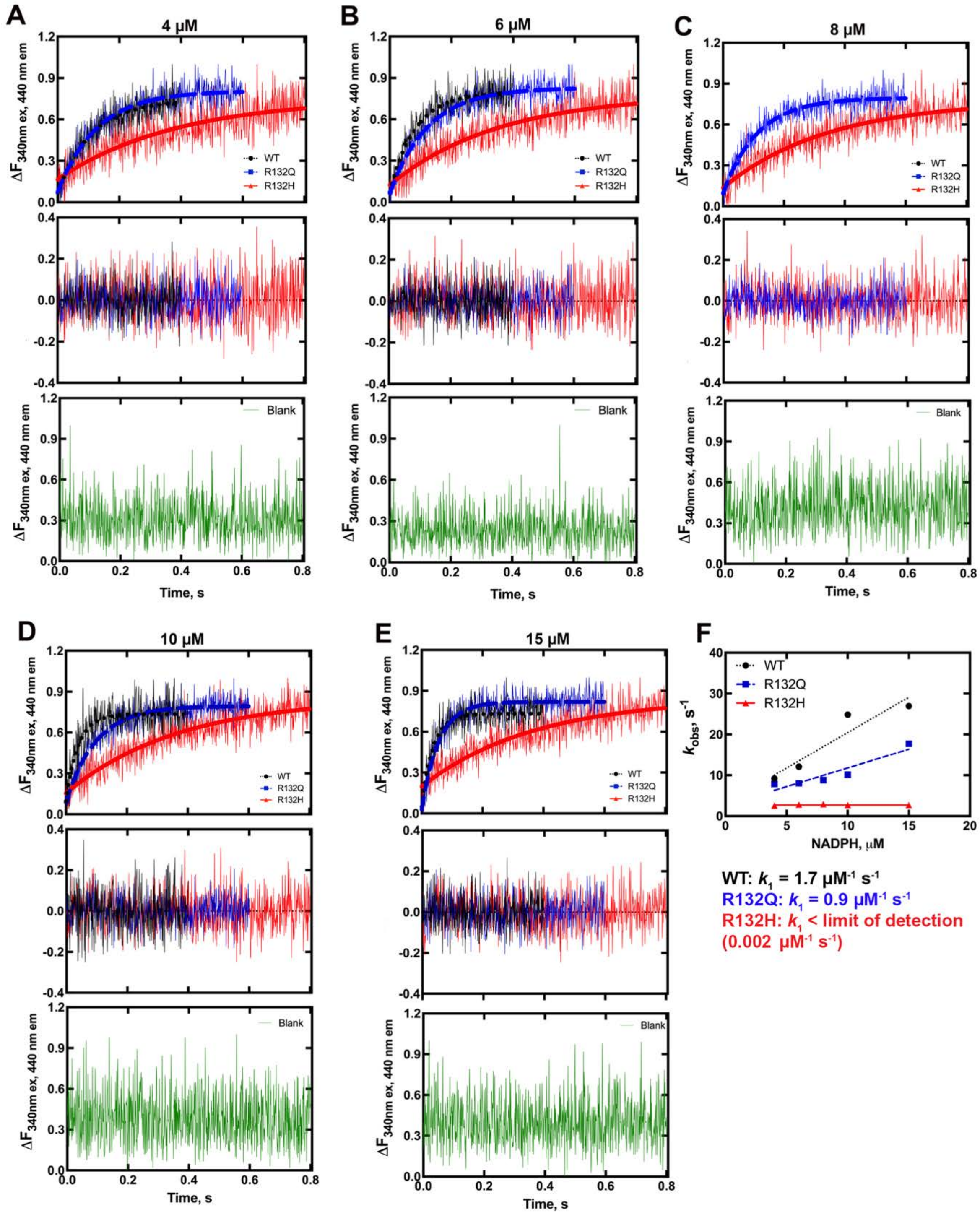

Supplementary Fig. 3. Rates of NADPH binding to IDH1 WT, R132Q, and R132H. A-E) NADPH binding was monitored at 10 ºC and 40% glycerol to slow the binding reaction. The change in fluorescence was fit to a single exponential equation (top plot) and residuals (middle plot) were obtained to assess goodness of fit. A control experiment lacking enzyme is shown in the bottom plot (in green). A) 4 μM NADPH. B) 6 μM NADPH. C) 8 μM NADPH. D) 10 μM NADPH. E) 15 μM NADPH. F) The k_{obs} values were plotted as a function of NADPH concentration to yield a linear progression, indicating one-step binding for NADPH. In all cases, a single protein preparation was used (biological replicate), with each trace representing an average of 10 technical replicates. The following kinetic parameters were measured: k_1 = 1.7 ± 0.4 μM^{-1} s^{-1}; 0.9 ± 0.2 μM^{-1} s^{-1}; and < the limit of detection (<0.002 ± 0.02 μM^{-1} s^{-1}) for WT (black), R132Q (blue), and R132H (red), respectively. Kinetic parameters were calculated and reported as +/- SEM resulting from deviation of the mathematical fit.

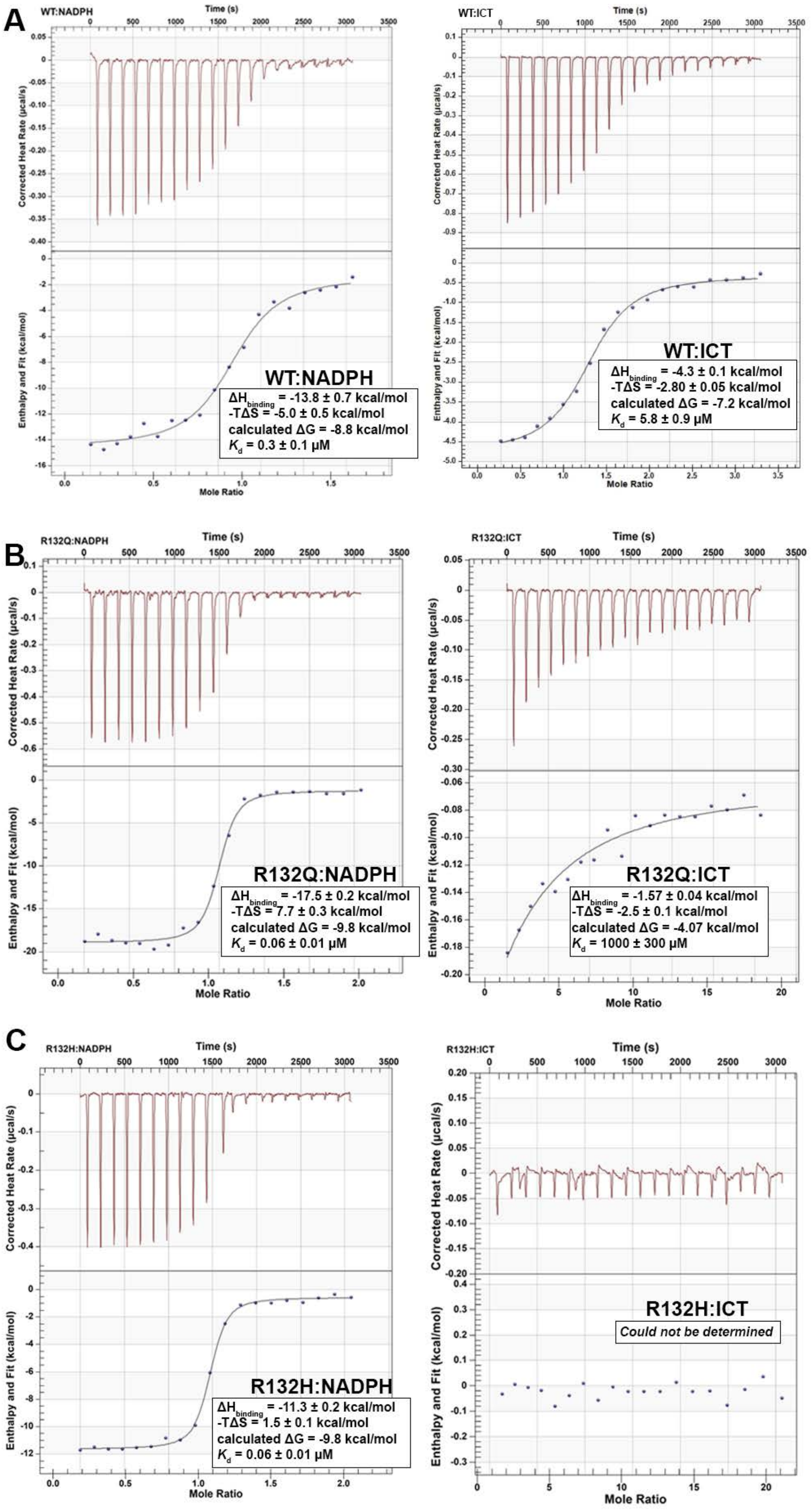

Supplementary Fig. 4. Binding affinities of IDH1 WT, R132Q and R132H for NADPH and ICT using isothermal titration calorimetry (ITC). A) IDH1 WT. B) IDH1 R132Q. C) IDH1 R132H. Kinetic parameters were calculated and reported as +/- SEM resulting from deviation of the mathematical fit.

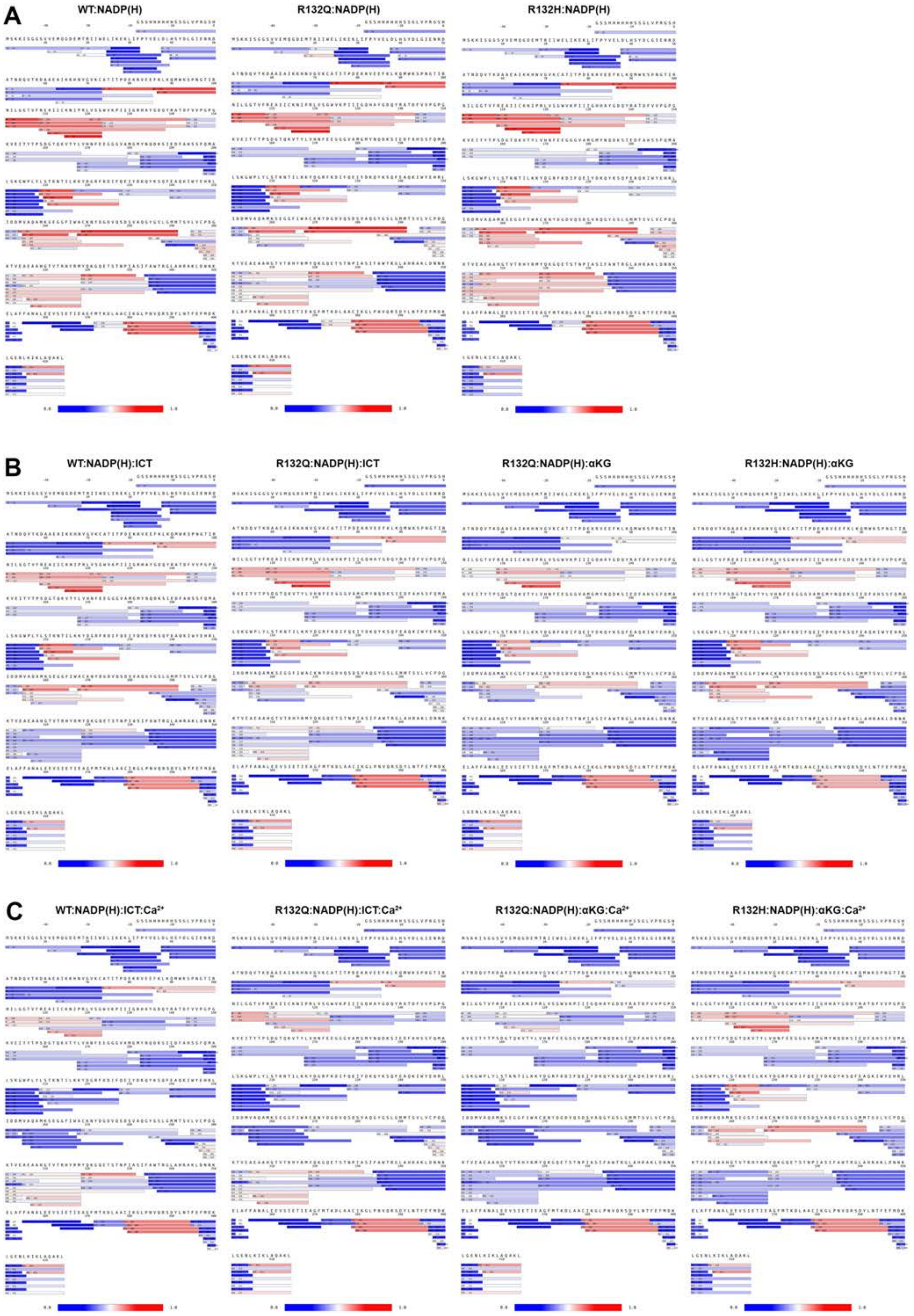
A
WT:NADP(H)
R132Q:NADP(H)
R132H:NADP(H)
B
WT:NADP(H):ICT
R132Q:NADP(H):ICT
R132Q:NADP(H):αKG
R132H:NADP(H):αKG
C
WT:NADP(H):ICT:Ca²⁺
R132Q:NADP(H):ICT:Ca²⁺
R132Q:NADP(H):αKG:Ca²⁺
R132H:NADP(H):αKG:Ca²⁺

Supplementary Fig. 5. Coverage maps with degree of deuterium uptake for IDH1 WT, R132Q, and R132H under three unique conditions. Deuterium uptake is shown as a gradient between highest uptake (red) to lowest uptake (blue). A) The WT, R132H, and R132Q binary form served as a baseline comparison. B) IDH1 WT and R132Q were treated with $NADP^+$ and ICT, and IDH1 R132Q and R132H were treated with NADPH and αKG (ternary complexes). C) WT and mutant IDH1 were treated as in B), except $CaCl_2$ was also included (quaternary complexes).

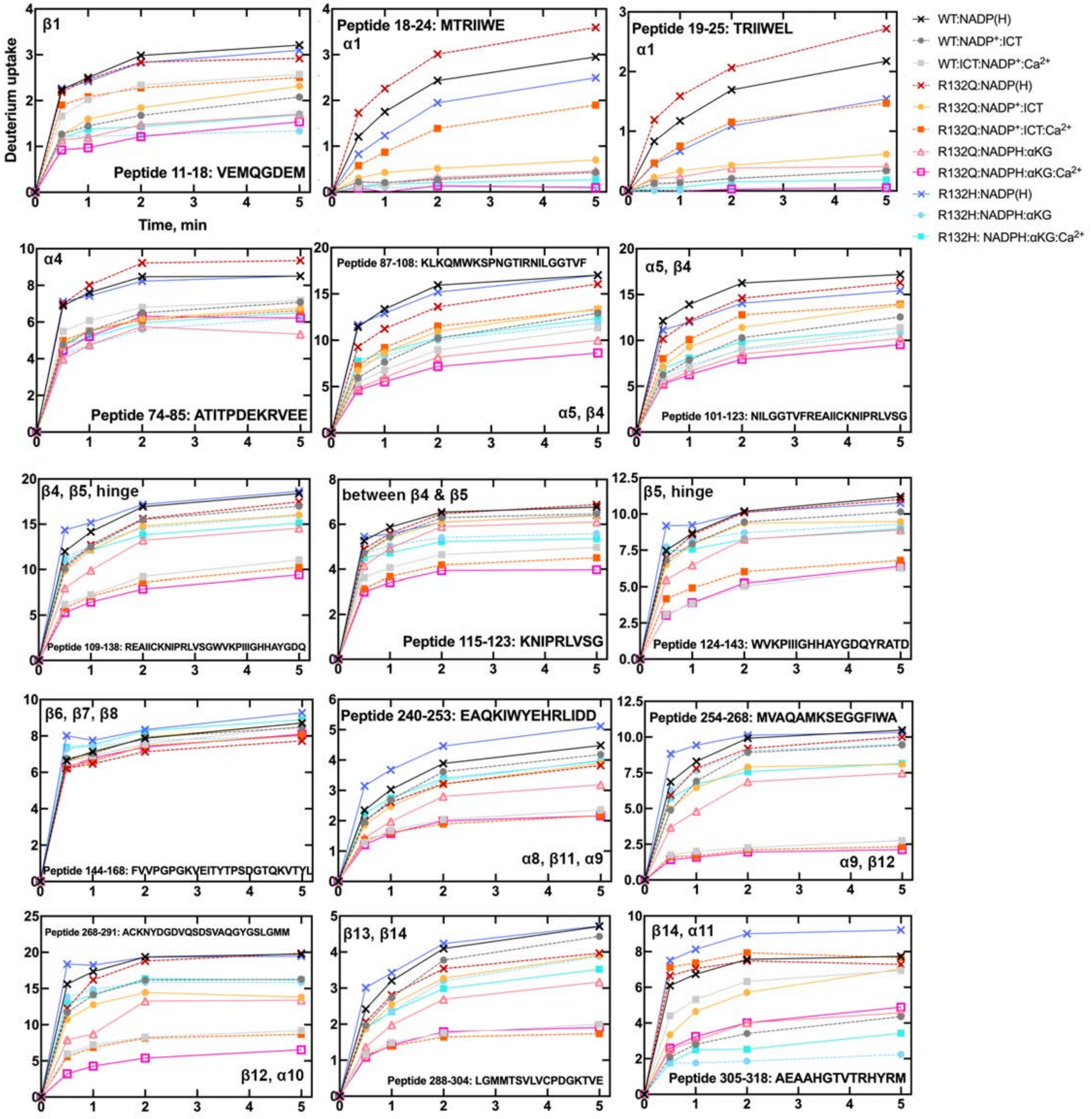

Supplementary Fig. 6. Deuterium uptake plots. Back-exchange corrected deuterium uptake was plotted against time for peptides of interest. Each point represents the mean of three technical replicates. Conditions are indicated in the plot legend. In the IDH1:NADP(H) conditions, any cofactors that bound during expression and purification were not removed, though no cofactor incubation step was added. Secondary structures associated with the peptides are indicated.

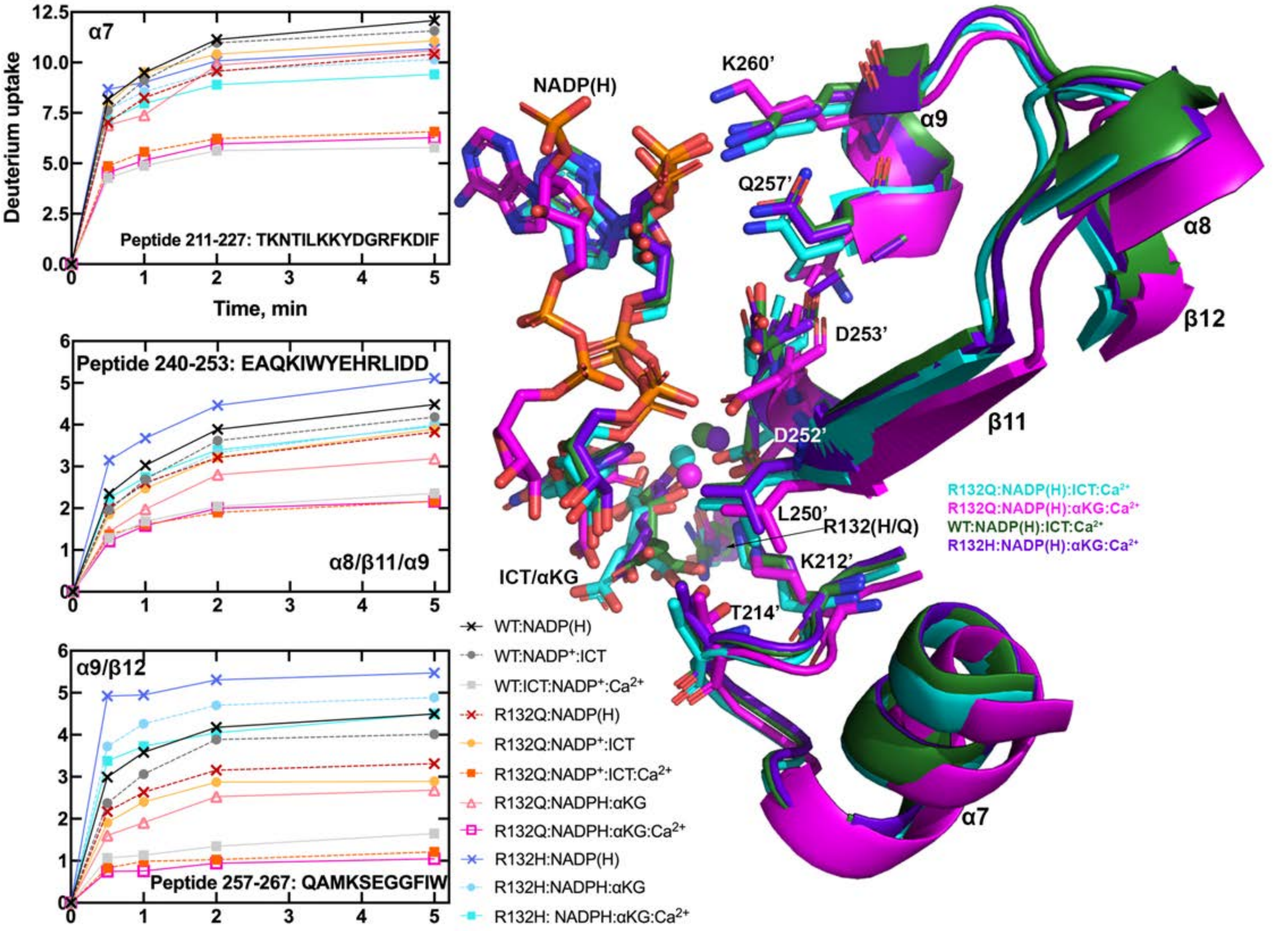

Supplementary Fig. 7. Peptides near the active site in IDH1 R132Q and WT have more amides that are exchanging at faster exchange rates than R132H upon binding substrates. Deuterium uptake plots for peptides containing chain B residues within 4 Å of the bound NADP(H) and ICT/αKG molecules (residues K212', T214', L250', D252', D253', Q257', and K260') are shown on the left. Each point represents the mean of three technical replicates. Secondary structure associated with these three peptides are shown on the right for ICT-bound R132Q and WT [1], and αKG-bound R132Q and R132H [2].

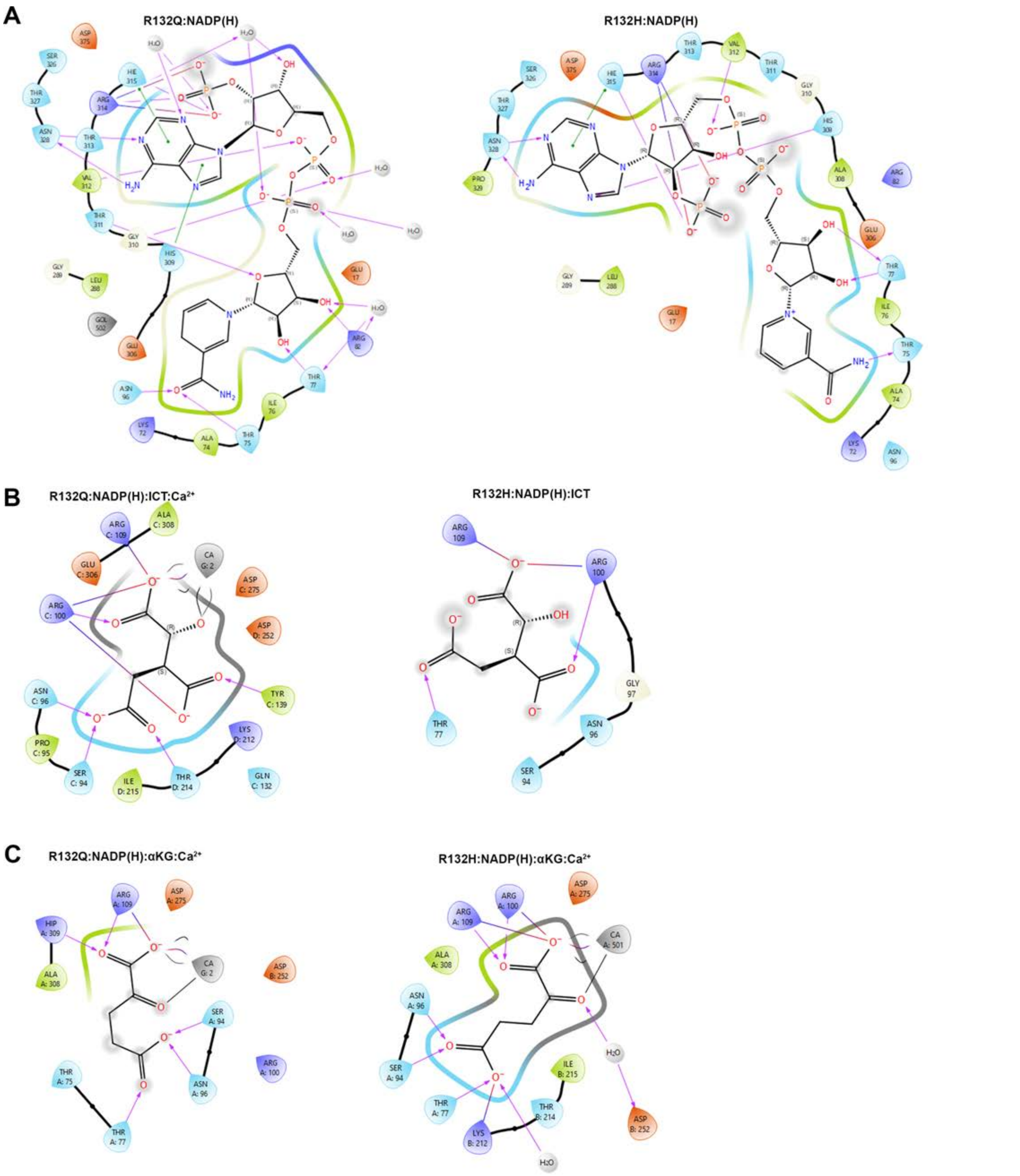

Supplementary Fig. 8. Ligand interaction diagrams highlighting hydrogen bonding interactions. In all cases, the residues are shown as guitar picks, with the pointed end signifying the side chain and the rounded end representing the backbone. A) Ligand interaction diagram between NADP(H) and IDH1 R132Q and waters in the cofactor binding pocket for the binary R132Q:NADP(H) structure. Hydrogen bonding interactions are shown with pink lines, while pi stacking interactions are shown with green. Residues within 4 Å of NADP(H) are

shown. In comparison, corresponding hydrogen bonding interactions of NADP(H) are also shown in the previously solved R132H:NADP(H) structure [3]. B) Ligand interaction diagram between ICT and IDH1 R132Q and Ca^{2+} in the R132Q binding pocket for the R132Q:NADP(H):ICT:Ca^{2+} structure. Hydrogen bonding interactions are shown with pink arrows while charge interactions are shown with red-blue lines. Residues within 4 Å of ICT are shown. In comparison, corresponding hydrogen bonding interactions of NADP(H) are also shown in R132H:NADP(H):ICT [3]. C) Ligand interaction diagram between αKG, Ca^{2+}, and IDH1 R132Q and in the R132Q binding pocket for the R132Q:NADP(H):αKG:Ca^{2+} structure (PDB 8VHB). Hydrogen bonding interactions are shown with pink arrows while charge interactions are indicated with red-blue lines. Residues within 4 Å of αKG are shown. In comparison, corresponding hydrogen bonding interactions of NADP(H) are also shown in the previously solved R132H:NADP(H):αKG:Ca^{2+} structure [2].

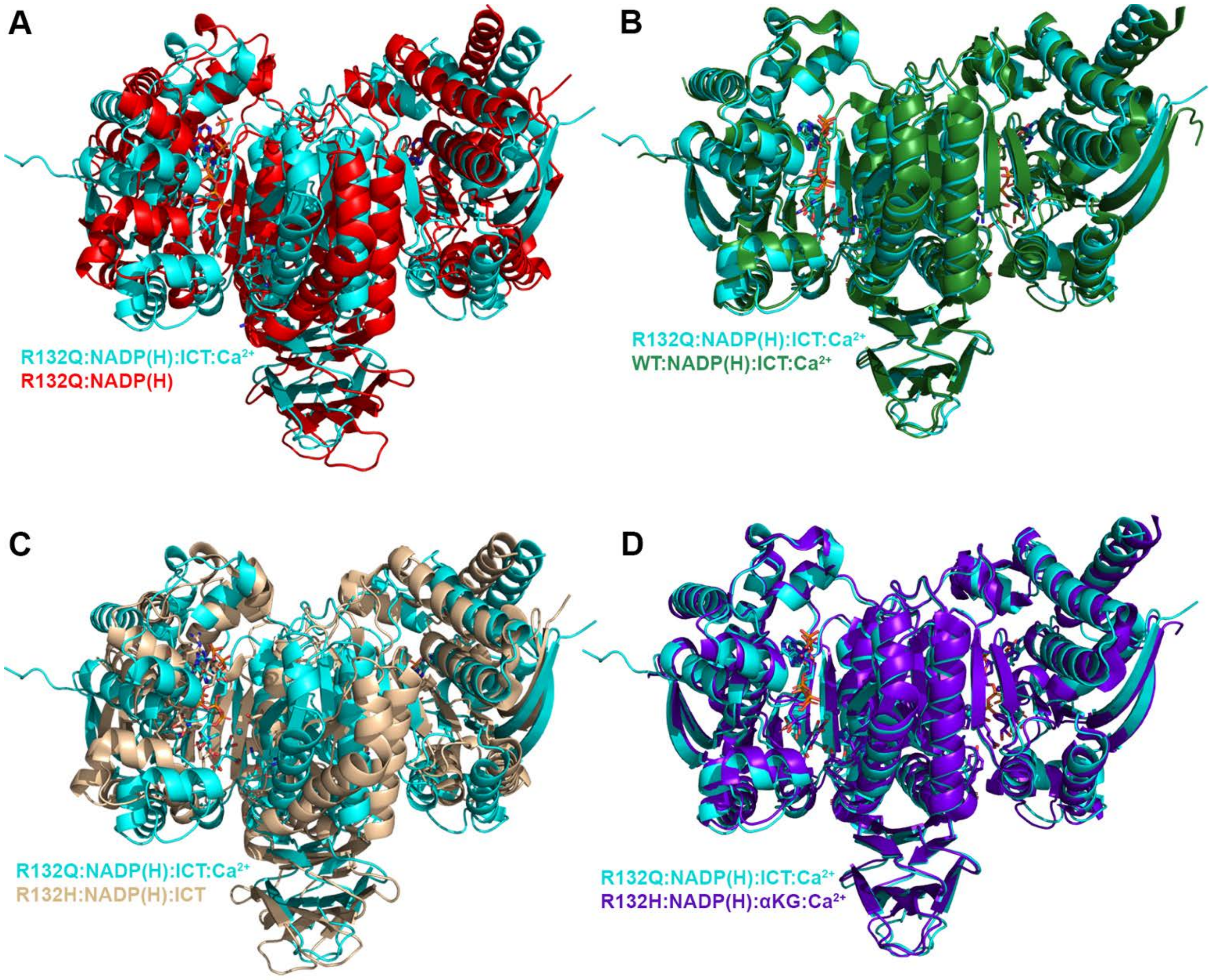

Supplementary Fig. 9. Structural alignments with IDH1 R132Q bound to ICT. R132Q:NADP(H):ICT:Ca^{2+} (cyan) is shown aligned via dimer to A) R132Q:NADP(H) (red); B) WT:NADP(H):ICT:Ca^{2+} [1] (dark green); C) R132H:NADP(H):ICT [3] (wheat); and D) R132H:NADP(H):αKG:Ca^{2+} [2] (dark purple).

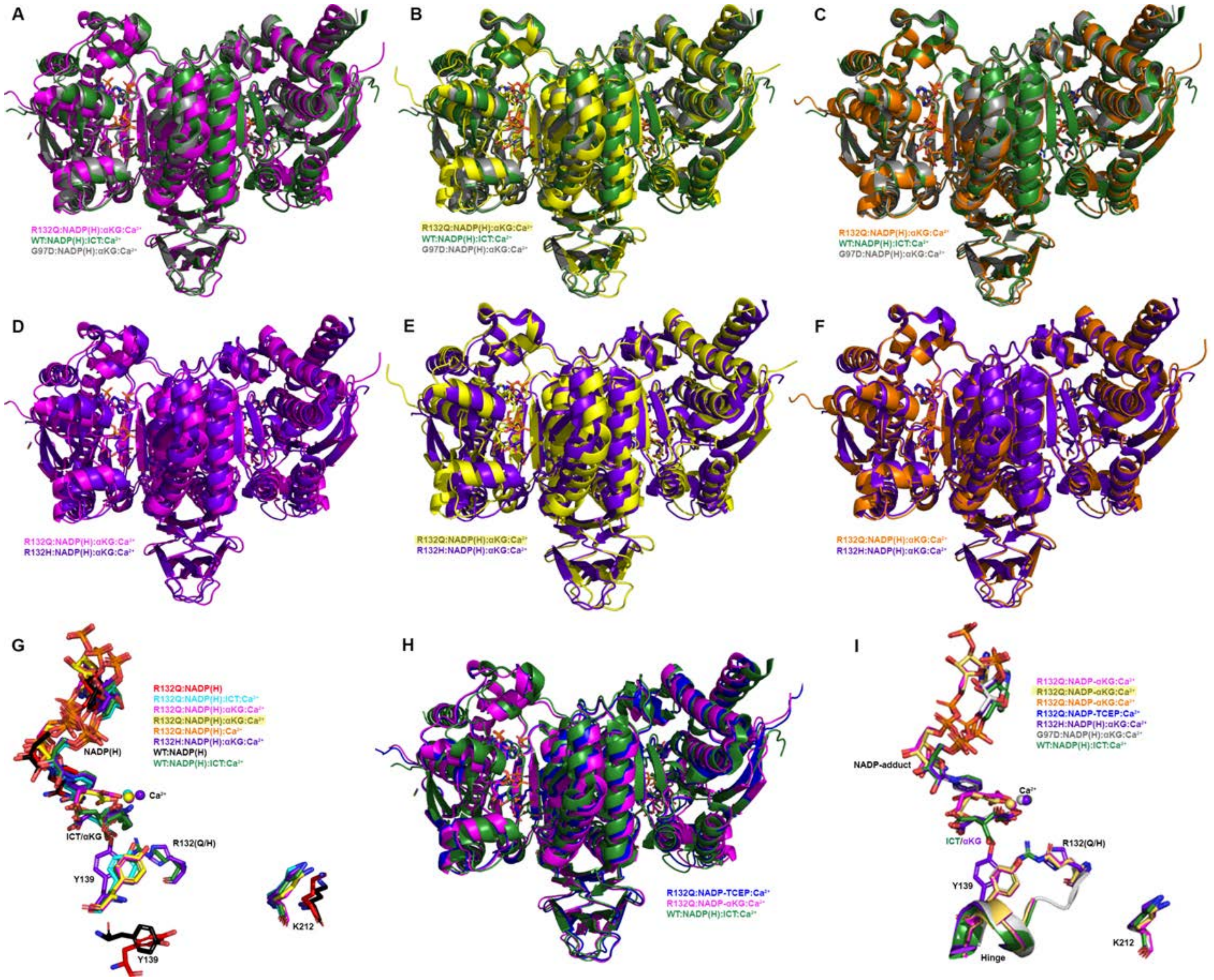

Supplementary Fig. 10. Structural alignments with IDH1 R132Q bound to αKG and NADP-adducts. In (A-C), structures of R132Q are shown aligned via their dimers to WT:NADP(H):ICT:Ca^{2+} [1] (dark green) and G97D:NADP(H):αKG:Ca^{2+} [2] (grey). A) R132Q:NADP(H):αKG:Ca^{2+}/R132Q:NADP-αKG:Ca^{2+} dimer (magenta). B) R132Q:NADP-αKG:Ca^{2+}/ R132Q:NADP(H):αKG:Ca^{2+} dimer 1 (yellow). C) R132Q:NADP-αKG:Ca^{2+}/R132Q:NADP(H):Ca^{2+} dimer 2 (orange). In (D-F), structures of R132Q are shown aligned via their dimers to R132H:NADP(H):αKG:Ca^{2+} [2] (purple). D) R132Q:NADP(H):αKG:Ca^{2+}/R132Q:NADP-αKG:Ca^{2+} dimer (magenta). E) R132Q:NADP-αKG:Ca^{2+}/ R132Q:NADP(H):αKG:Ca^{2+} dimer 1 (yellow). F) R132Q:NADP-αKG:Ca^{2+}/R132Q:NADP(H):Ca^{2+} dimer 2 (orange). G) Monomers of non-adduct-containing monomers were aligned, with substrates, R132X, Ca^{2+}, and catalytic residues highlighted. Shown are features of R132Q:NADP(H) (red), R132Q:NADP(H):ICT:Ca^{2+} (cyan), R132Q:NADP(H):αKG:Ca^{2+} (magenta), R132Q:NADP(H):αKG:Ca^{2+} from dimer 1 (yellow), R132Q:NADP(H):Ca^{2+} from dimer 2 (orange), R132H:NADP(H):αKG:Ca^{2+} [2] (purple), WT:NADP(H) [1] (black), and WT:NADP(H):ICT:Ca^{2+} [1] (dark green).

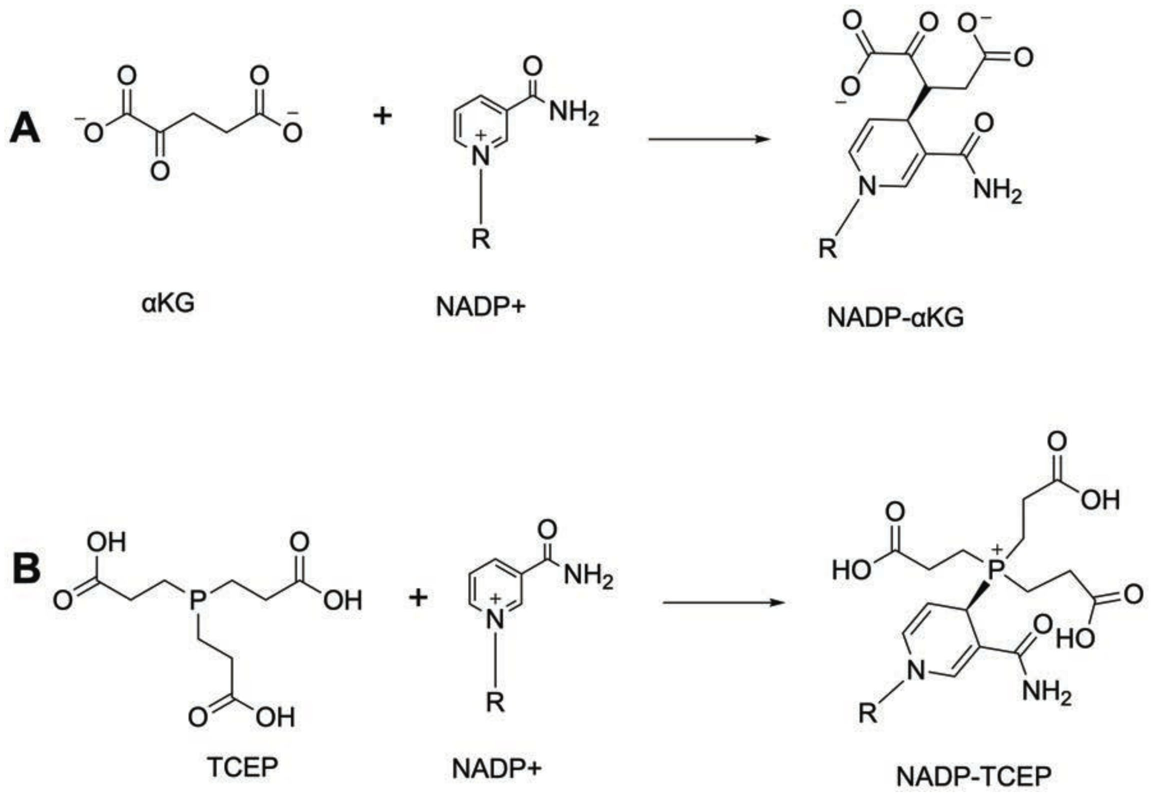

Supplementary Fig. 11. Observed adduct formation upon IDH1 R132Q crystallization. A) NADP-αKG adduct formation. We propose that crystallographic conditions (likely thiocyanate[4]), resulted in the oxidation of NADPH to generate $NADP^+$ required to form the adduct. B) NADP-TCEP adduct formation.

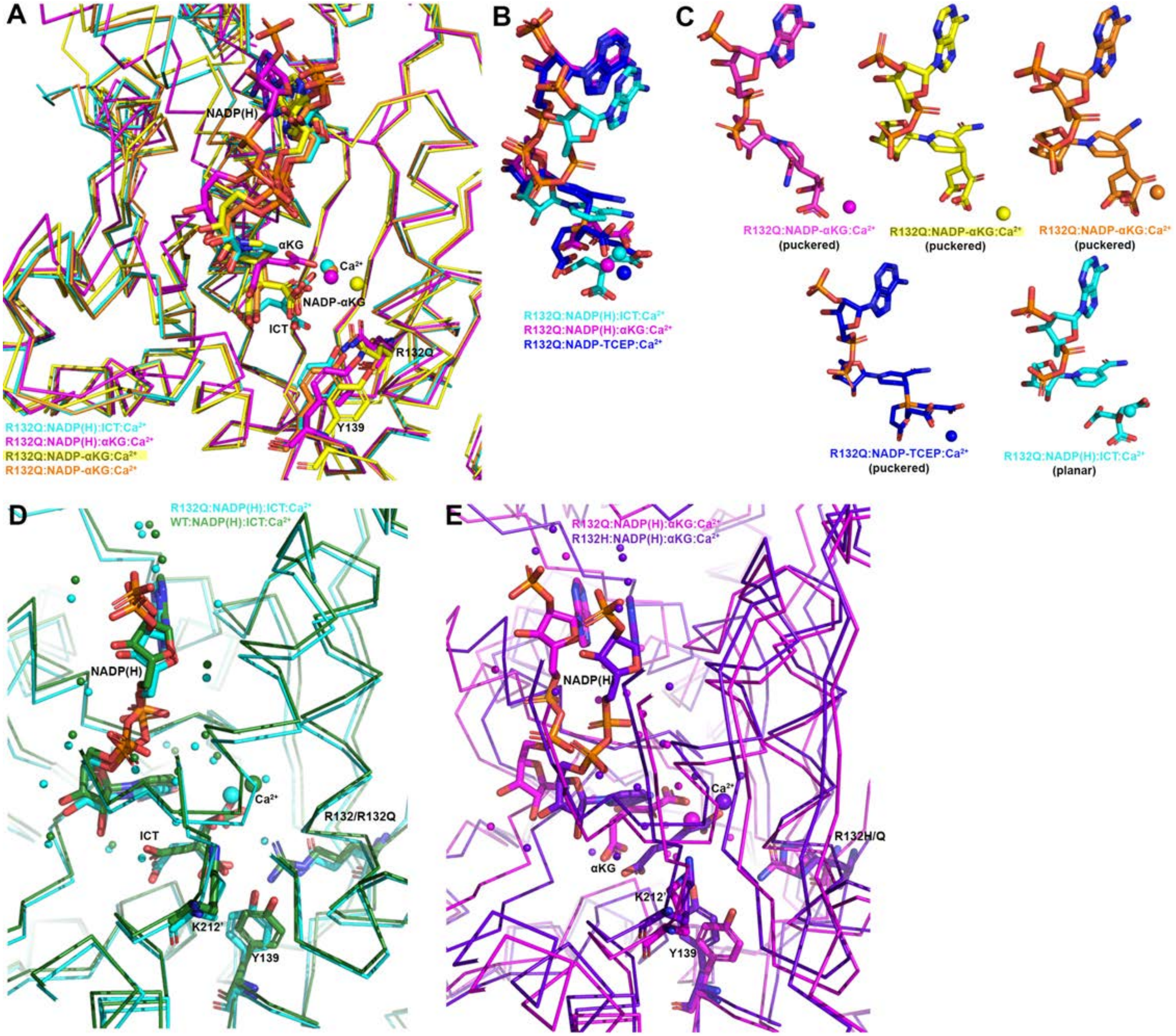

Supplementary Fig. 12. IDH1 R132Q structures vary in substrate binding location, degree of nicotinamide ring puckering, and active site waters. A) Monomer-based alignment of the R132Q:NADP-αKG:Ca^{2+} monomer (orange) that aligns with R132Q:NADP(H):ICT:Ca^{2+}(cyan), and the R132Q:NADP-αKG:Ca^{2+} monomer (yellow) that aligns as a transition between R132Q:NADP(H):ICT:Ca^{2+} and R132Q:NADP(H):αKG:Ca^{2+} (magenta). B) Only the NADP(H) and ICT or αKG, or the NADP-adduct, with Ca^{2+}, are highlighted. Though the nicotinamide ring in the R132Q:NADPH:αKG:Ca^{2+} structure could not be confidently modeled, comparisons of the planar nicotinamide ring of R132Q:NADP(H):ICT:Ca^{2+} versus the puckered rings of the NADP-adducts are featured in these monomer-based alignments of R132Q:NADP(H):ICT:Ca^{2+}, R132Q:NADPH:αKG:Ca^{2+}, and R132Q:NADP-TCEP:Ca^{2+} (dark blue). C) Just the NADP(H), ICT, and Ca^{2+}, or NADP-adduct and Ca^{2+} are highlighted to show a planar or puckered nicotinamide ring. In (D-E), dimer-based alignments are shown. D) Water molecules (smaller spheres) within 3.5 Å of the substrates are highlighted in ICT-containing structures. E) Water molecules (smaller spheres) within 3.5 Å of the substrates are highlighted in αKG-containing structures.

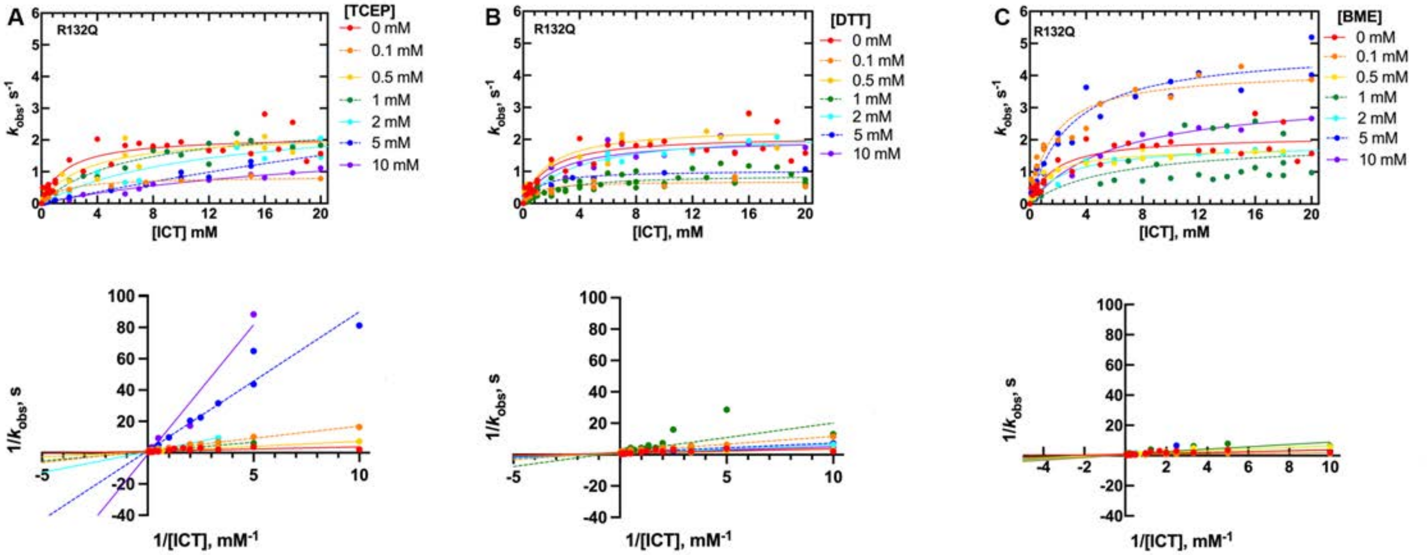

Supplemental Fig. 13. TCEP treatment competitively inhibits the forward reaction catalyzed by IDH1 R132Q. Steady-state kinetic parameters of the conventional reaction by IDH1 R132Q were measured as a function of varying ICT concentration upon challenge with one of three reducing agents. Top: Three protein preparations were used to measure the observed rate constants (k_{obs}), which were determined from the linear portion of plots of substrate concentration versus time, with each point representing a single replicate. Bottom: Lineweaver-Burk analysis was performed by plotting $1/k_{obs}$ *vs* 1/[ICT]. A) IDH1 R132Q catalysis upon increasing concentrations with TCEP. Only treatment with TCEP showed notable, dose-dependent inhibition, with features of competitive inhibition. B) IDH1 R132Q catalysis upon increasing concentrations with DTT. C) IDH1 R132Q catalysis upon increasing concentrations with BME. A table of the kinetic parameters is shown in Supplementary Table 4.

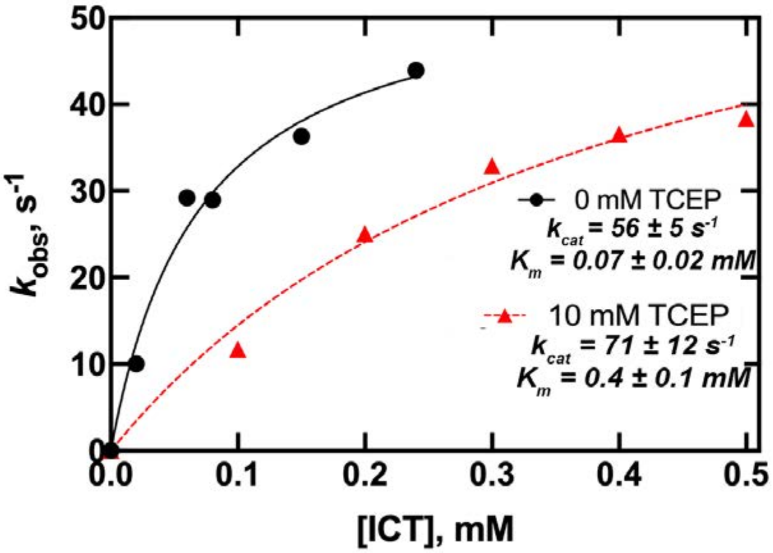

Supplementary Fig. 14. TCEP treatment has less of an effect on IDH1 WT catalysis. While conversion of ICT to αKG by IDH1 R132Q is associated with a 19-fold increase in K_m in the presence of 10 mM TCEP, we see only a 6-fold increase for IDH1 WT. These points represent single replicates from one protein preparation. Kinetic parameters were calculated and reported as +/- SEM resulting from deviation of the mathematical fit. The following kinetic parameters were measured for IDH1 WT with 0 mM TCEP treatment (black circles): $k_{cat,\ ICT\rightarrow\alpha KG}$ = 56 ± 5 s^{-1}; $K_{m,\ ICT}$ = 0.07 ± 0.03 mM; and with 10 mM TCEP treatment (red triangles): $k_{cat,\ ICT\rightarrow\alpha KG}$ = 71 ± 12 s^{-1}; $K_{m,\ ICT}$ = 0.4 ± 0.1 mM.

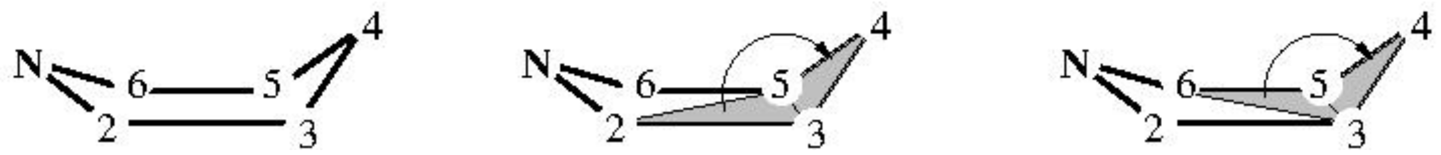

Supplementary Fig. 15. Atom labeling and choice of dihedral angles for determination of deviation from planarity of atom C4. The positions of the N atom 1 and the opposite C atom 4 are referenced to the plane defined by the roughly coplanar atoms 2, 3, 5, and 6. The average of the dihedral angles 2-3-5-4 and 6-3-5-4 shown here is subtracted from 180º to yield $\Delta\theta_{\mathrm{C}}$ as a metric for the deviation from planarity of C4, while the average of 3-2-6-1 and 5-2-6-1 subtracted from 180º is used to calculate $\Delta\theta_{\mathrm{N}}$ for N1.

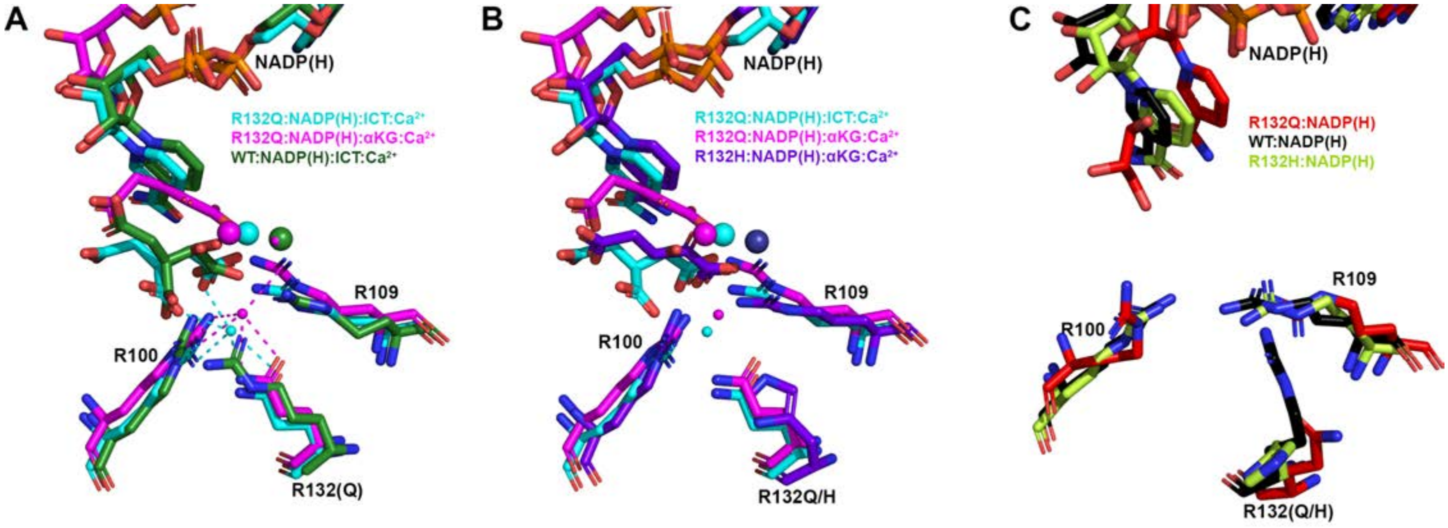

Supplementary Fig. 16. IDH1 R132Q employs an active site water molecule to mimic polar interactions normally provided by residue R132. Dimer-based alignments of the active site are shown that highlight residues R100, R109, and R132(Q/H), which play an important role in hydrogen binding to ICT in the absence of mutation at residue R132. A) Both ICT-bound (cyan) and αKG-bound (magenta) R132Q structures featured an active site water (small spheres) that helped coordinate ICT, though this water molecule was absent in the quaternary WT structure [1] (dark green). A second water molecule localized to the position of the calcium ion (large spheres) in the IDH1 WT structure. B) The quaternary R132H structure [2] (purple) did not contain a coordinating active site water. C) The binary NADP(H) bound forms of R132Q (red), R132H [3] (light green), and WT [1] (black) lacked this active site water.

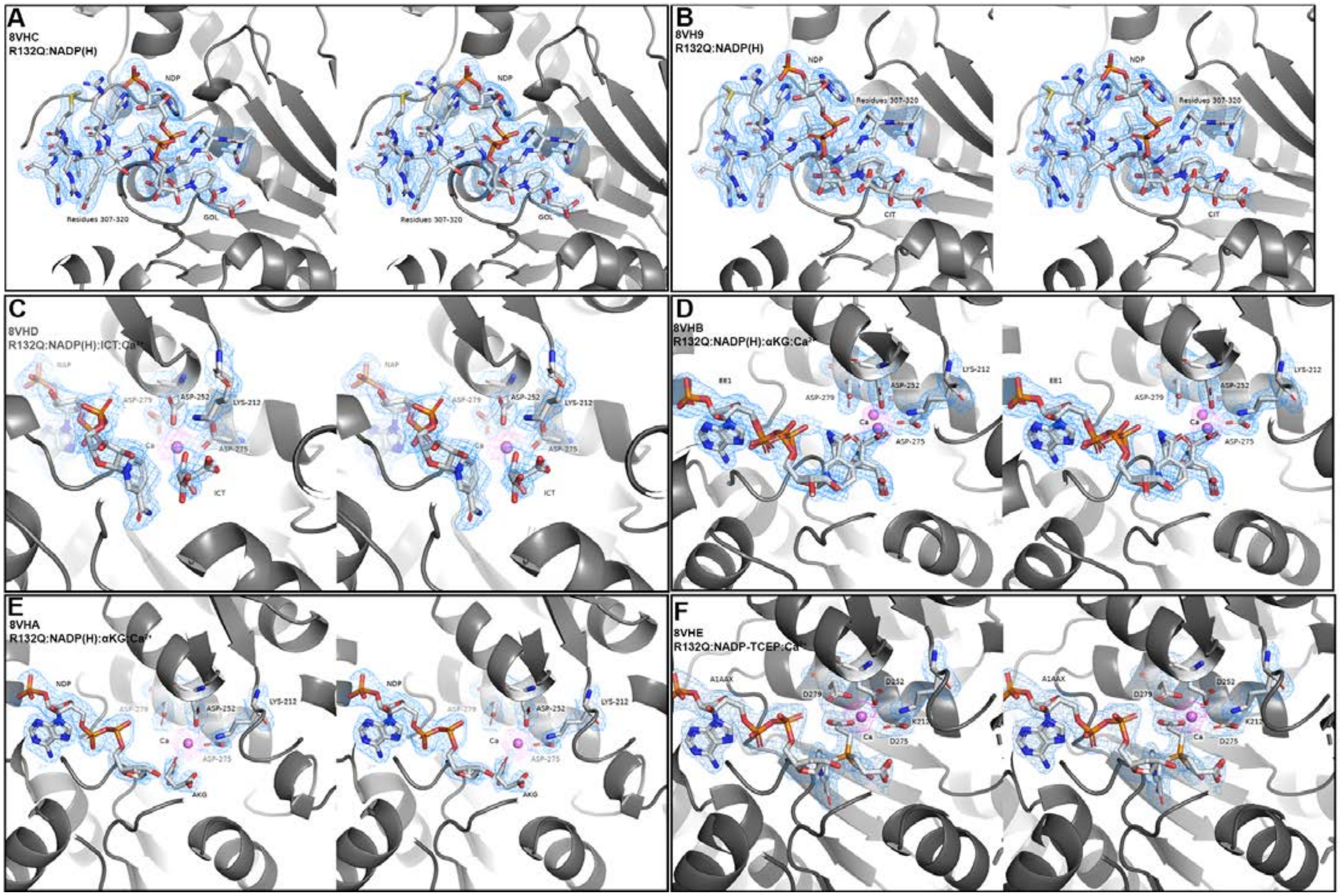

Supplementary Fig. 17. Stereo-image of a section of the electron density map for the IDH1 R132Q structures. A) R132Q:NADP(H) (PDB 8VHC), with residues 307-320, NADP(H) (NDP), and glycerol (GOL) highlighted. B) R132Q:NADP(H) (PDB 8VH9), with residues 307-320, NADP(H) (NDP), and citrate (CIT) highlighted. C) R132Q:NADP(H):ICT:Ca^{2+} (PDB 8VHD), with D279, D252, K212, D275, NADP(H) (NAP), Ca^{2+}, and ICT highlighted. D) R132Q:NADP(H):αKG:Ca^{2+} (PDB 8VHB), with D279, D252, K212, D275, NADP- αKG adduct (EE1), and Ca^{2+} highlighted. E) R132Q:NADP(H):αKG:Ca^{2+} (PDB 8VHA). F) R132Q:NADP-TCEP:Ca^{2+} (PDB 8VHE), with D279, D252, K212, D275, NADP- TCEP adduct (A1AAX), and Ca^{2+} highlighted.

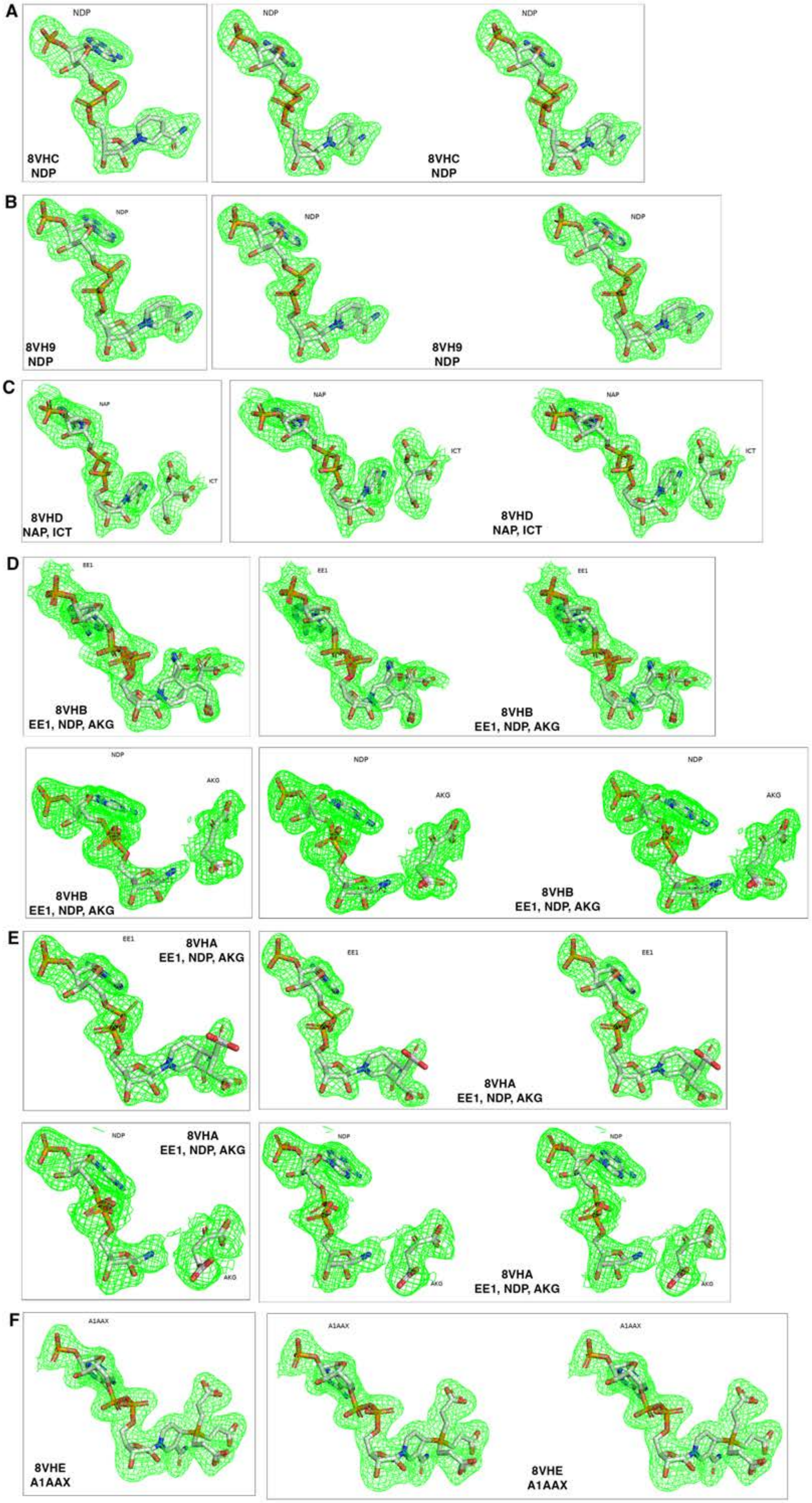
A
NDP
8VHC
NDP
B
8VH9
C
NAP
ICT
8VHD
NAP, ICT
D
EE1
8VHB
EE1, NDP, AKG
AKG
E
8VHA
F
A1AAX
8VHE

Supplementary Fig. 18. mFo-DFc omit maps for ligands. All of the mFo-DFc omit maps are contoured at 3 sigma shown with the single image view on the left and wall-eyed stereo image view on the right. A) Ligands in PDB code 8VHC. B) Ligands in PDB code 8VH9. C) Ligands in PDB code 8VHD. D) Ligands in PDB code 8VHB. E) Ligands in PDB code 8VHA. F) Ligands in PDB code 8VHE.

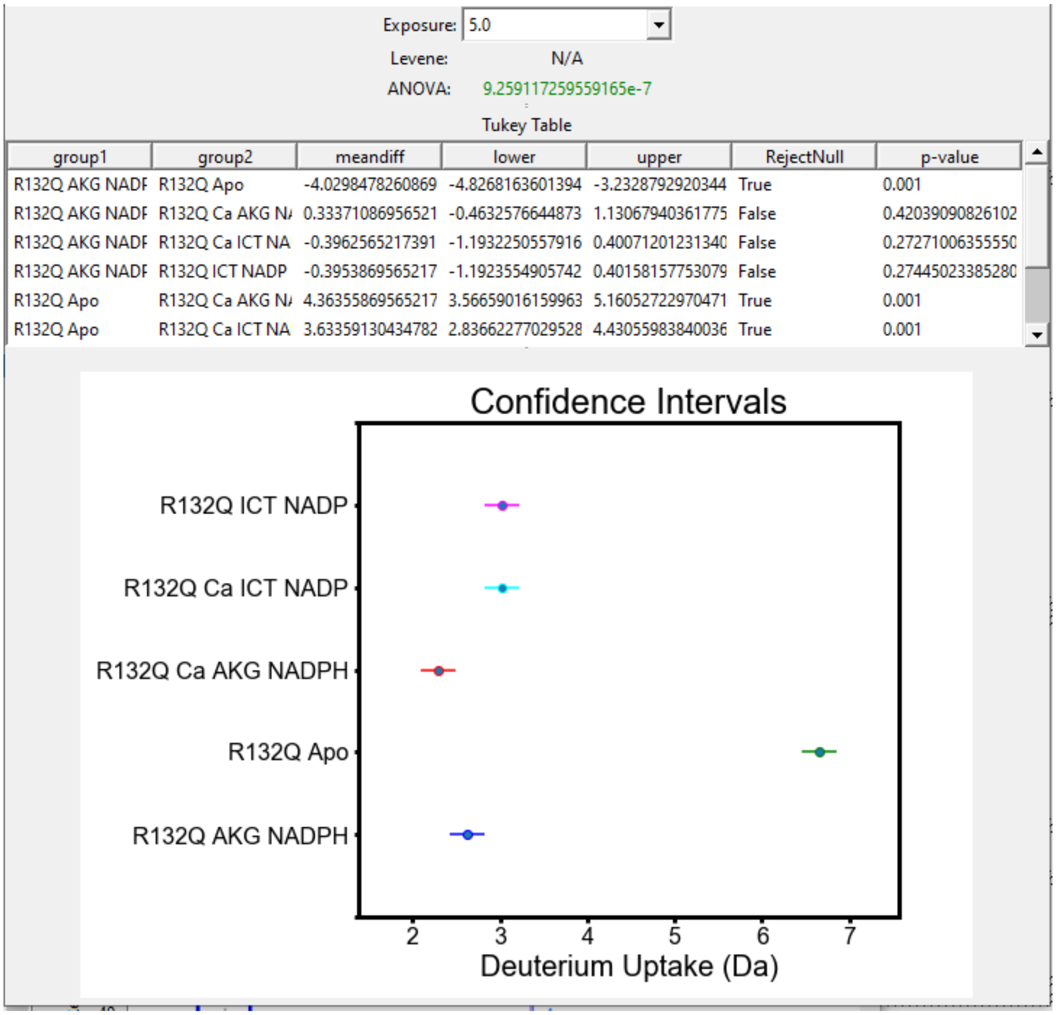

group1	group2	meandiff	lower	upper	RejectNull	p-value
R132Q AKG NADF	R132Q Apo	-4.0298478260869	-4.8268163601394	-3.2328792920344	True	0.001
R132Q AKG NADF	R132Q Ca AKG N/	0.33371086956521	-0.4632576644873	1.13067940361775	False	0.42039090826102
R132Q AKG NADF	R132Q Ca ICT NA	-0.3962565217391	-1.1932250557916	0.40071201231340	False	0.27271006355550
R132Q AKG NADF	R132Q ICT NADP	-0.3953869565217	-1.1923554905742	0.40158157753079	False	0.27445023385280
R132Q Apo	R132Q Ca AKG N/	4.36355869565217	3.56659016159963	5.16052722970471	True	0.001
R132Q Apo	R132Q Ca ICT NA	3.63359130434782	2.83662277029528	4.43055983840036	True	0.001

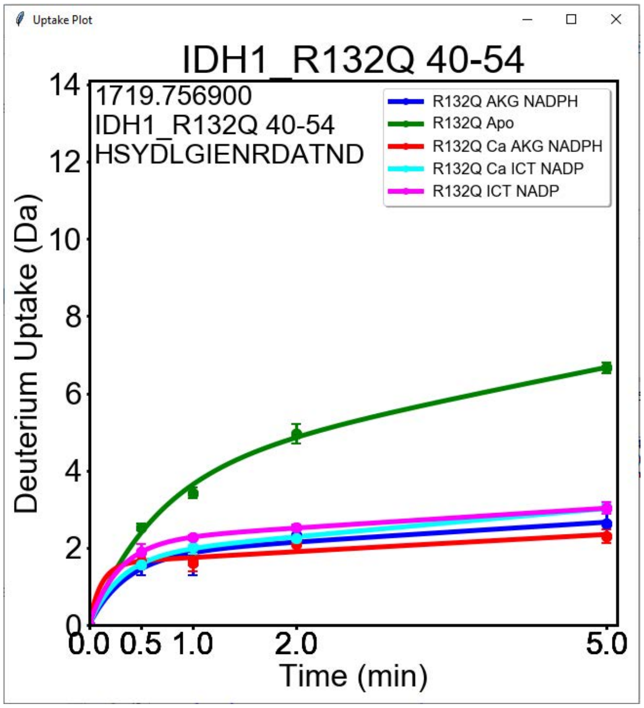

Supplementary Fig. 19. Statistical panel generated from DECA. The statistical panel generated from DECA for the 5 min time point for the peptide shown in the associated uptake plot for a representative peptide (IDH1 R132Q peptide 40-54).

Supplementary Table 1. Active site and back cleft measurements assessing open versus closed conformations. By convention, active site clefts are measured as the distance between residues 76 in chain A and 250' in chain B, and back clefts are measured as the distance between residues 199 to 342 in chain A. Active, closed conformations are associated with small active site clefts and large back clefts.

PDB ID	IDH1 complex (monomer/monomer listed if applicable)	I76 to L250' active site cleft distance, Å	I76' to L250 active site cleft distance, Å	M199 to H342 back cleft distance, Å	M199' to H342' back cleft distance, Å
8VHC	R132Q:NADP(H) [a]	14.7	16.9	9.1	8.9
8VH9	R132Q:NADP(H) [b]	14.2	17.1	8.7	8.4
8VHD	R132Q:NADP(H):ICT:Ca^{2+}/ R132Q:NADP(H):Ca^{2+}	8.8 (ICT)[c]	10.4 (no ICT)	10.1 (ICT)	10.3 (no ICT)
8VHB	R132Q:NADP(H):αKG:Ca^{2+}/ R132Q:NADP-αKG:Ca^{2+}	10.6 (αKG)	11.7 (adduct)	9.8 (αKG)	9.5 (adduct)
8VHA	R132Q:NADP(H):αKG:Ca^{2+}/ R132Q:NADP-αKG:Ca^{2+}	11.3 (αKG)	12.2 (adduct)	10.2 (αKG)	10.0 (adduct)
8VHA	R132Q:NADP-αKG:Ca^{2+}/ R132Q:NADP(H):Ca^{2+}	8.7 (adduct)	11.8 (no αKG)	9.7 (adduct)	10.6 (no αKG)
8VHE	R132Q:NADP-TCEP: Ca^{2+}	11.8	11.3	9.6	10.7
1T09[1]	WT:NADP(H)	17.6	14.8	8.3	9.0
1T0L[1]	WT:NADP(H):ICT:Ca^{2+}	8.8	8.6	11.1	11.1
4L03[2]	G97D:NADP(H):αKG:Ca^{2+}	8.6	8.7	10.9	11.1
3MAR[3]	R132H:NADP(H)	17.8	15.1	8.3	8.4
3MAP[3]	R132H:NADP(H):ICT	16.1	14.1	7.7	8.0
4KZO[2]	R132H:NADP(H):αKG:Ca^{2+}	8.4	8.6	10.9	11.1

[a] Condition crystallized in sulfate
[b] Condition crystallized in citrate
[c] Dimers containing monomers with different molecules in the active site are indicated in the second column, and measurements associated with each monomer are identified by listing the active site molecule present in parenthesis.

Supplementary Table 2. Relative energies and free energies of model $NADP^+$ and TCEP for binding, from B3LYP/pc-1 calculations. The final energies and free energies reported were calculated by adding the counterpoise corrections to the energies of the geometries optimized with solvation.

**Species**	**Relative E_0[a] (kcal mol^{-1})**	**Relative E (298K)[b] (kcal mol^{-1})**	**Relative G (kcal mol^{-1})**
NADP + TCEP	0	0	0
NADP-TCEP TS	2.5	-2.2	24.6
NADP-TCEP adduct	-4.3	-9.4	20.0

[a] Relative electronic energy, including solvation and counterpoise corrections but excluding zero-point and thermal contributions.
[b] Relative energy, including solvation, counterpoise, zero-point, and thermal contributions.

Supplementary Table 3. Dihedral angle measurements. Using the angles shown in Supplementary Fig. 15, dihedral angles of the nicotinamide ring for NADP(H) or NADP-adduct molecules bound to IDH1 R132Q are shown. A sign convention has been applied such that if $\Delta\theta_C$ and $\Delta\theta_N$ have the same sign, the two corners of the ring bend away each other in chair fashion, whereas opposite signs indicate a boat-like conformation.

**Species**	**Structural details**	**$\Delta\theta_C$ (º)**	**$\Delta\theta_N$ (º)**	**Conformation**
NADP(H)	R132Q:NADP(H), PDB 8VHC (chain A)	2.4	-4.1	Planar
NADP(H)	R132Q:NADP(H), PDB 8VHC (chain B)	4.8	-7.6	Planar
NADP(H)	R132Q:NADP(H):ICT:Ca^{2+}, PDB 8VHD (ICT monomer)	0.4	0.2	Planar
NADP(H)	R132Q:NADP(H):ICT:Ca^{2+}, PDB 8VHD (non-ICT monomer)	0.3	0.1	Planar
NADP-αKG<br><br>*DFT-calculated values:*	R132Q:NADP(H):αKG:Ca^{2+}, PDB 9VHB (adduct monomer opposite αKG monomer)	25.0<br><br>*29*	-25.6<br><br>*-14*	Boat-like
NADP-αKG	R132Q:NADP(H):αKG:Ca^{2+}, PDB 8VHA (adduct monomer opposite αKG monomer)	26.1	-3.0	Boat-like
NADP-αKG	R132Q:NADP(H):αKG:Ca^{2+}, PDB 8VHA (adduct monomer opposite non-αKG monomer)	15.4	-14.1	Boat-like
NADP-TCEP<br><br>*DFT-calculated values:*	R132Q:NADP-TCEP:Ca^{2+}, PDB 8VHE (chain A)	29.2<br><br>*25*	-1.1<br><br>*-11*	Boat-like
NADP-TCEP	R132Q:NADP-TCEP:Ca^{2+}, PDB 8VHE (chain B)	26.0	9.3	Chair-like

Supplementary Table 4. Steady-state kinetic parameters for conversion of ICT to αKG by IDH1 R132Q upon challenge with reducing agents. TCEP treatment resulted in inhibition of the conventional reaction catalyzed by IDH1 R132Q. We did not observe any change in activity upon 10 mM TCEP treatment for the neomorphic reaction catalyzed by IDH1 R132H (k_{cat} = 1.0 ± 0.03, K_m = 0.42 ± 0.05) and by IDH1 R132Q (k_{cat} = 2.11 ± 0.07, K_m = 0.21 ± 0.04), which use NADPH as a substrate rather than $NADP^+$. Kinetic parameters were calculated and reported as +/- SEM.

[Reducing agent]	$k_{cat,\ ICT \rightarrow \alpha KG}$ (s^{-1})	$K_{M,\ ICT}$ (mM)	$k_{cat}/K_{M,\ ICT \rightarrow \alpha KG}$ ($mM^{-1}s^{-1}$)
**[TCEP]**			
0 mM	2.1 ± 0.1	1.7 ± 0.5	1.2 ± 0.4
0.1 mM	0.84 ± 0.04	1.6 ± 0.3	0.53 ± 0.09
0.5 mM	2.3 ± 0.3	4 ± 1	0.7 ± 0.3
1 mM	2.7 ± 0.3	6 ± 2	0.4 ± 0.1
2 mM	3.1 ± 0.9	14 ± 8	0.2 ± 0.2
5 mM	8 ± 4	83 ± 45	0.09 ± 0.07
10 mM	2.7 ± 0.5	32 ± 9	0.09 ± 0.03
**[DTT]**			
0 mM	2.1 ± 0.1	1.7 ± 0.5	1.2 ± 0.4
0.1 mM	0.69 ± 0.05	0.9 ± 0.2	0.80 ± 0.02
0.5 mM	2.4 ± 0.2	1.8 ± 0.8	1.3 ± 0.6
1 mM	0.90 ± 0.06	2.1 ± 0.5	0.42 ± 0.09
2 mM	2.2 ± 0.2	4 ± 1	0.62 ± 0.2
5 mM	1.03 ± 0.06	1.1 ± 0.2	1.0 ± 0.2
10 mM	2.0 ± 0.1	2.2 ± 0.7	0.9 ± 0.3
**[BME]**			
0 mM	2.1 ± 0.1	1.7 ± 0.5	1.2 ± 0.4
0.1 mM	4.2 ± 0.2	1.6 ± 0.3	2.5 ± 0.5
0.5 mM	1.75 ± 0.07	1.3 ± 0.2	1.3 ± 0.2
1 mM	2.0 ± 0.7	6 ± 7	0.34 ± 0.4
2 mM	1.87 ± 0.08	2.5 ± 0.5	0.76 ± 0.2
5 mM	4.9 ± 0.3	2.8 ± 0.5	1.7 ± 0.3
10 mM	3.4 ± 0.3	5.9 ± 1.4	0.6 ± 0.2

Supplementary Table 5. HDX-MS parameters.

Data Set	WT:NADP(H)	WT:NADP(H):ICT	WT:NADP(H):ICT:Ca^{2+}
HDX reaction details	50 mM Tris, 100 mM NaCl, pD= 7.85 @ 4 °C	50 mM Tris, 100 mM NaCl, pD= 7.85 @ 4 °C	50 mM Tris, 100 mM NaCl, pD= 7.85 @ 4 °C
HDX time course (min)	0.5, 1, 2, 5	0.5, 1, 2, 5	0.5, 1, 2, 5
HDX control samples	Disordered section of WT NT protein	Disordered section of WT NT protein	Disordered section of WT NT protein
Back-exchange (mean/IQR)	42%/5%	42%/5%	42%/5%
# of Peptides	112	112	112
Sequence coverage	99.8%	99.8%	99.8%
Average peptide length/Redundancy	15.9/ 4.12	15.9/ 4.12	15.9/ 4.12
Replicates (biological or technical)	3 (technical)	3 (technical)	3 (technical)
Repeatability	0.046 (average standard deviation)	0.047 (average standard deviation)	0.053 (average standard deviation)
Significant differences in HDX (Δ HDX > X D)	0.25D (99% CI)	0.25D (99% CI)	0.25D (99% CI)

Data Set	R132H:NADP(H)	R132H:NADP(H):αKG	R132H:NADP(H):αKG:Ca^{2+}
HDX reaction details	50 mM Tris, 100 mM NaCl, pD= 7.85 @ 4 °C	50 mM Tris, 100 mM NaCl, pD= 7.8 5@ 4 °C	50 mM Tris, 100 mM NaCl, pD= 7.85 @ 4 °C
HDX time course (min)	0.5, 1, 2, 5	0.5, 1, 2, 5	0.5, 1, 2, 5
HDX control samples	Disordered section of WT NT protein	Disordered section of WT NT protein	Disordered section of WT NT protein
Back-exchange (mean / IQR)	31%/5%	31%/5%	31%/5%
# of Peptides	112	112	112
Sequence coverage	99.8%	99.8%	99.8%
Average peptide length / Redundancy	15.9/ 4.12	15.9/ 4.12	15.9/ 4.12
Replicates (biological or technical)	3 (technical)	3 (technical)	3 (technical)
Repeatability	0.089 (average standard deviation)	0.105 (average standard deviation)	0.079 (average standard deviation)
Significant differences in HDX (Δ HDX > X D)	0.25D (99% CI)	0.25D (99% CI)	0.25D (99% CI)

Data Set	R132Q:NADP(H)	R132Q:NADP(H):ICT	R132Q:NADP(H):ICT:Ca^{2+}	R132Q:NADP(H):αKG	R132Q:NADP(H):αKG:Ca^{2+}
HDX reaction details	50 mM Tris, 100 mM NaCl, pD= 7.85 @ 4 °C	50 mM Tris, 100 mM NaCl, pD= 7.85 @ 4 °C	50 mM Tris, 100 mM NaCl, pD= 7.85 @ 4 °C	50 mM Tris, 100 mM NaCl, pD= 7.85 @ 4 °C	50 mM Tris, 100 mM NaCl, pD= 7.85 @ 4 °C
HDX time course (min)	0.5, 1, 2, 5	0.5, 1, 2, 5	0.5, 1, 2, 5	0.5, 1, 2, 5	0.5, 1, 2, 5
HDX control samples	Disordered section of WT NT protein	Disordered section of WT NT protein	Disordered section of WT NT protein	Disordered section of WT NT protein	Disordered section of WT NT protein
Back-exchange (mean / IQR)	51% / 5%	51% / 5%	51% / 5%	51% / 5%	51% / 5%
# of Peptides	112	112	112	112	112
Sequence coverage	99.8%	99.8%	99.8%	99.8%	99.8%
Average peptide length / Redundancy	15.9/4.12	15.9/4.12	15.9/4.12	15.9/4.12	15.9/4.12
Replicates (biological or technical)	3 (technical)	3 (technical)	3 (technical)	3 (technical)	3 (technical)
Repeatability	0.060 (average standard deviation)	0.064 (average standard deviation)	0.061 (average standard deviation)	0.144 (average standard deviation)	0.069 (average standard deviation)
Significant differences in HDX (Δ HDX > X D)	0.25D (99% CI)	0.25D (99% CI)	0.25D (99% CI)	0.25D (99% CI)	0.25D (99% CI)

Supplementary Table 6. Crystallography parameters. Statistics for the highest-resolution shell are shown in parentheses.

Data collection	**R132Q:NADP(H) (sulfate condition)**	**R132Q:NADP(H) (citrate condition)**	**R132Q:NADP(H):ICT:Ca^{2+}**	**R132Q:NADP(H):αKG: Ca^{2+}**	**R132Q:NADP(H):αKG: Ca^{2+}**	**R132Q:NADP-TCEP:Ca^{2+}**
PDB code	8VHC	8VH9	8VHD	8VHB	8VHA	8VHE
Space Group	P 43 21 2	P 43 21 2	P 1 21 1	P 1 21 1	P 1 21 1	P 1 21 1
Cell Dimensions a, b, c (Å) α, β, γ (°)	82.884 82.884 303.926 90.00 90.00 90.00	81.085 81.085 306.136 90.00 90.00 90.00	84.408 103.894 108.272 90.00 98.54 90.00	84.404 105.807 109.782 90.00 98.44 90.00	83.821 104.86 107.711 90.00 98.19 90.00	84.298 107.348 109.941 90.00 99.19 90.00
Resolution (Å)	2.44	2.13	2.38	1.89	2.28	2.16
Observations	80846 (7762)	518814 (53176)	142446 (13835)	288375 (28444)	162883 (15717)	304736
Unique reflections	40453 (3891)	58370 (5722)	72946 (6517)	148111 (14777)	82712 (8073)	101523 (10184)
Source	APS 24-ID-E	APS 24-ID-E	SSRL Beamline 12-1	SSRL Beamline 12-1	SSRL Beamline 12-1	APS 24-ID-E
Wavelength (Å)	.979180	.979180	.97946	.97946	.97946	.979180
R-merge	0.02171 (0.4383)	0.08036 (1.172)	0.08184 (0.44)	0.02985 (0.3369)	0.04588 (0.3619)	0.05029 (0.2894)
<I/σ(I)>	22.37 (1.77)	18.29 (2.02)	5.28 (2.29)	12.71 (2.34)	10.65 (2.20)	13.0 (2.57)
Completeness	99.60 (98.78)	99.91 (99.98)	96.88 (87.89)	97.12 (97.04)	98.28 (96.82)	98.01 (98.76)
Refinement						
Resolution range (Å)	72.76 - 2.44 (2.527 - 2.44)	76.53 - 2.13 (2.206 - 2.13)	39.18 - 2.38 (2.465 - 2.38)	39.63 - 1.89 (1.958 - 1.89)	39.15 - 2.28 (2.361 - 2.28)	83.22 - 2.16 (2.237 - 2.16)
Protein residues	794	795	1650	1653	1649	1660
Ligand atoms	186	199	237	448	334	327
Water Atoms	75	343	801	1069	462	933
R-work	0.1922 (0.3187)	0.1756 (0.2863)	0.1694 (0.2144)	0.1680 (0.2685)	0.1673 (0.2185)	0.1732 (0.2291)
R-free	0.2398 (0.3678)	0.2153 (0.3172)	0.2199 (0.2885)	0.1723 (0.2692)	0.2222 (0.2925)	0.2218 (0.2770)
Wilson B-Factor	58.0	41.1	22.9	29.1	35.6	34.1
RMS (bonds)	0.004	0.010	0.002	0.008	0.009	0.005
RMS (angles)	0.59	1.02	0.42	0.92	0.91	0.67
Clash Score	12.42	7.39	3.29	9.69	8.02	3.36
Ramachandran plot						
Favored (%)	95.78	95.68	96.59	96.84	96.59	96.49
Allowed (%)	4.09	4.32	3.29	3.04	3.35	3.51
Outliers (%)	0.13	0.00	0.12	0.12	0.06	0.00

Supplementary References

1. Xu, X. *et al.* Structures of human cytosolic NADP-dependent isocitrate dehydrogenase reveal a novel self-regulatory mechanism of activity. *J Biol Chem* **279**, 33946–57 (2004).
2. Rendina, A. R. *et al.* Mutant IDH1 enhances the production of 2-hydroxyglutarate due to its kinetic mechanism. *Biochemistry* **52**, 4563–77 (2013).
3. Yang, B., Zhong, C., Peng, Y., Lai, Z. & Ding, J. Molecular mechanisms of 'off-on switch' of activities of human IDH1 by tumor-associated mutation R132H. *Cell Res* **20**, 1188–200 (2010).
4. Hogg, D. M. & Jago, G. R. The oxidation of reduced nicotinamide nucleotides by hydrogen peroside in the presence of lactoperoxidase and thiocyanate, iodide or bromide. *Biochem J* **117**, 791–797 (1970).
