# Supplementary material for: Active site remodeling in tumor-relevant IDH1 mutants drives distinct kinetic features and potential resistance mechanisms
Source: Nat Commun. 2024 May 6;15:3785. doi: 10.1038/s41467-024-48277-2 (PMC11074275; doi:10.1038/s41467-024-48277-2)
Supplement: Supplementary file 3 — Description of Additional Supplementary Files [file 41467_2024_48277_MOESM3_ESM.pdf]

Description of Additional Supplementary Files

File Name: Supplementary Data 1
Description: HDX-MS IDH1 WT data

File Name: Supplementary Data 2
Description: HDX-MS IDH1 R132Q data

File Name: Supplementary Data 3
Description: HDX-MS IDH1 R132H data
