# Supplementary material for: Active site remodeling in tumor-relevant IDH1 mutants drives distinct kinetic features and potential resistance mechanisms
Source: Nat Commun. 2024 May 6;15:3785. doi: 10.1038/s41467-024-48277-2 (PMC11074275; doi:10.1038/s41467-024-48277-2)
Supplement: Supplementary file 7 — Reporting Summary [file 41467_2024_48277_MOESM7_ESM.pdf]

nature portfolio

Corresponding author(s): Christal D. Sohl

Last updated by author(s): Apr 23, 2024

Reporting Summary

Nature Portfolio wishes to improve the reproducibility of the work that we publish. This form provides structure for consistency and transparency in reporting. For further information on Nature Portfolio policies, see our Editorial Policies and the Editorial Policy Checklist.

Statistics

For all statistical analyses, confirm that the following items are present in the figure legend, table legend, main text, or Methods section.

n/a	Confirmed	
☐	☒	The exact sample size (n) for each experimental group/condition, given as a discrete number and unit of measurement
☐	☒	A statement on whether measurements were taken from distinct samples or whether the same sample was measured repeatedly
☒	☐	The statistical test(s) used AND whether they are one- or two-sided<br>*Only common tests should be described solely by name; describe more complex techniques in the Methods section.*
☒	☐	A description of all covariates tested
☒	☐	A description of any assumptions or corrections, such as tests of normality and adjustment for multiple comparisons
☐	☒	A full description of the statistical parameters including central tendency (e.g. means) or other basic estimates (e.g. regression coefficient) AND variation (e.g. standard deviation) or associated estimates of uncertainty (e.g. confidence intervals)
☒	☐	For null hypothesis testing, the test statistic (e.g. F, t, r) with confidence intervals, effect sizes, degrees of freedom and P value noted<br>*Give P values as exact values whenever suitable.*
☒	☐	For Bayesian analysis, information on the choice of priors and Markov chain Monte Carlo settings
☒	☐	For hierarchical and complex designs, identification of the appropriate level for tests and full reporting of outcomes
☒	☐	Estimates of effect sizes (e.g. Cohen's d, Pearson's r), indicating how they were calculated

Our web collection on statistics for biologists contains articles on many of the points above.

Software and code

Policy information about availability of computer code

Data collection	HDX-MS data were collected using HDxDirector software v1.0.4.0, Waters nanoAcquity UPLC Auxillary Solvent Manager v1.50.2601, Waters nanoAcquity UPLC Binary Solvent Manager v1.50.1327, MassLynx 4.1 SCN917, and ProteinLynx global server (PLGS) v3.0.<br>Crystallographic data were collected using XDS v6/30/23.
Data analysis	HDX-MS data were analyzed using DynamX v3.0.0 and DECA v116.<br>Kinetics experiments were plotted and analyzed using Prism v10.<br>Density functional theory (DFT) calculations were made using Gaussian 16.<br>For crystallographic data analysis, XDS, Phenix v1.20, and Coot v1.1 were used.<br>ITC data were analyzed using Nanoanalyze by TA Instruments.

For manuscripts utilizing custom algorithms or software that are central to the research but not yet described in published literature, software must be made available to editors and reviewers. We strongly encourage code deposition in a community repository (e.g. GitHub). See the Nature Portfolio guidelines for submitting code & software for further information.

Data

Policy information about availability of data

All manuscripts must include a data availability statement. This statement should provide the following information, where applicable:

- Accession codes, unique identifiers, or web links for publicly available datasets
- A description of any restrictions on data availability
- For clinical datasets or third party data, please ensure that the statement adheres to our policy

Crystallographic data and protein structure coordinates have been deposited with the Protein Data Bank (PDB) public repository: 8VHC [https://www.wwpdb.org/pdb?id=pdb_00008vhc] (Fo-wing), 8VH9 [https://www.wwpdb.org/pdb?id=pdb_00008vh9] (Fo-wing), 8VHD [https://www.wwpdb.org/pdb?id=pdb_00008vhd] (Fo-wing), 8VHB [https://www.wwpdb.org/pdb?id=pdb_00008vhb] (Fo-wing), 8VHA [https://www.wwpdb.org/pdb?id=pdb_00008vha] (Fo-wing), and 8VHE [https://www.wwpdb.org/pdb?id=pdb_00008vhe] (Fo-wing). Previously solved structures are also available: 1T0L [https://www.wwpdb.org/pdb?id=pdb_00001t0l] (Fo-wing), 4KZO [https://www.wwpdb.org/pdb?id=4kzo] (Fo-wing), 6PAY [https://www.wwpdb.org/pdb?id=pdb_00006pay] (Fo-wing), 1T09 [https://www.wwpdb.org/pdb?id=pdb_00001t09] (Fo-wing) and 4UMX [https://www.wwpdb.org/pdb?id=pdb_00004umx] (Fo-wing).

Output files from the computational work are available at the ioChem-BD database [https://doi.org/10.19061/iochem-bd-6-320].

HDX-MS data can be found at the MassIVE FTP server MSV000094158 [https://massive.ucsd.edu/ProteoSAFe/dataset.jsp?task=d24eb2fc5c0a4a2d9437dc1598212530]

Supplementary files are included (supplementary figures and tables in File 1, HDX-MS data in Files 2-4). Source data is also included.

Additional information and requests for resources and reagents should be directed for fulfillment by the corresponding author Christal D. Sohl (csohl@sdsu.edu).

Research involving human participants, their data, or biological material

Policy information about studies with human participants or human data. See also policy information about sex, gender (identity/presentation), and sexual orientation and race, ethnicity and racism.

Reporting on sex and gender	No sexed or gendered organisms were used in this work (only E. coli).
Reporting on race, ethnicity, or other socially relevant groupings	No sexed or gendered organisms were used in this work (only E. coli).
Population characteristics	No sexed or gendered organisms were used in this work (only E. coli).
Recruitment	No sexed or gendered organisms were used in this work (only E. coli).
Ethics oversight	No sexed or gendered organisms were used in this work (only E. coli).

Note that full information on the approval of the study protocol must also be provided in the manuscript.

Field-specific reporting

Please select the one below that is the best fit for your research. If you are not sure, read the appropriate sections before making your selection.

☒ Life sciences ☐ Behavioural & social sciences ☐ Ecological, evolutionary & environmental sciences

For a reference copy of the document with all sections, see nature.com/documents/nr-reporting-summary-flat.pdf

Life sciences study design

All studies must disclose on these points even when the disclosure is negative.

Sample size	Sample size is not appropriate in this study as we are not working with populations/pools. We ensure rigor in the number of our Michaelis-Menten plots by monitoring the standard, though we use standard error to assess deviation from fit and increase the number of points to decrease this to < 10%.
Data exclusions	Data were excluded from one experimental set with the R132Q kinetic measurements upon discovering an error in protein quantitation. Otherwise, no data were excluded.
Replication	For our steady-state kinetic experiments, we used either 2 or 3 biological replicates (protein preparations) as indicated in the manuscript plus two technical replicates to measure kobs values, and even with the same protein preparations, values were measured on different days and often by different students. Pre-steady-state plots are an average of 4 technical replicates for single turnover experiments, 10 for NADPH binding), and residual analysis was used to assess goodness of fit. All attempts at replication were successful here.
Randomization	We run kinetic samples not in increasing or decreasing order of concentration, but instead select concentrations at random.

Blinding	Anonymization (ie blinding) was not relevant in this study as we were limited to biophysical measurements -- the protein identity would be the only thing we can anonymize here, and this is experimentally impractical. The experimenter would know what mutant they were working with as they needed to know whether to test the conventional or neomorphic conditions, which protein to crystallize, etc.

Reporting for specific materials, systems and methods

We require information from authors about some types of materials, experimental systems and methods used in many studies. Here, indicate whether each material, system or method listed is relevant to your study. If you are not sure if a list item applies to your research, read the appropriate section before selecting a response.

Materials & experimental systems

n/a	Involved in the study
☒	☐ Antibodies
☒	☐ Eukaryotic cell lines
☒	☐ Palaeontology and archaeology
☒	☐ Animals and other organisms
☒	☐ Clinical data
☒	☐ Dual use research of concern
☒	☐ Plants

Methods

n/a	Involved in the study
☒	☐ ChIP-seq
☒	☐ Flow cytometry
☒	☐ MRI-based neuroimaging

Plants

Seed stocks	*Report on the source of all seed stocks or other plant material used. If applicable, state the seed stock centre and catalogue number. If plant specimens were collected from the field, describe the collection location, date and sampling procedures.*
Novel plant genotypes	*Describe the methods by which all novel plant genotypes were produced. This includes those generated by transgenic approaches, gene editing, chemical/radiation-based mutagenesis and hybridization. For transgenic lines, describe the transformation method, the number of independent lines analyzed and the generation upon which experiments were performed. For gene-edited lines, describe the editor used, the endogenous sequence targeted for editing, the targeting guide RNA sequence (if applicable) and how the editor was applied.*
Authentication	*Describe any authentication procedures for each seed stock used or novel genotype generated. Describe any experiments used to assess the effect of a mutation and, where applicable, how potential secondary effects (e.g. second site T-DNA insertions, mosiacism, off-target gene editing) were examined.*
